# Supplementary material for: An Unexpected Reaction of Isodehydracetic Acid with Amines in the Presence of 1-Ethyl-3-(3-dimethylaminopropyl) Carbodiimide Hydrochloride Yields a New Type of β-Enaminones
Source: Molecules. 2020 May 2;25(9):2131. doi: 10.3390/molecules25092131 (PMC7249165; doi:10.3390/molecules25092131)
Supplement: Supplementary file 1 [file molecules-25-02131-s001.pdf]

Supplementary Materials

An Unexpected Reaction of Isodehydracetic Acid with Amines in the Presence of 1-Ethyl-3-(3-dimethylaminopropyl) Carbodiimide Hydrochloride Yields a New Type of β-Enaminones

Delong Wang*,[1] and Hui Shi [2]

[1] Department of pharmaceutical engineering, Shanxi Agricultural University, Taigu 030801, Shanxi, China. *rizhaoalong@163.com*; Tel: +86 354-6286398
[2] State Key Laboratory of Coal Conversion, Institute of Coal Chemistry, Chinese Academy of Sciences, Taiyuan 030001, Shanxi, China. *shihui@sxicc.ac.cn*
* Corresponding author.

Table of contents:

1. NMR spectra……………………………………………………………….………………………*S2*

2. Computational details…………………………………………………………….………………………*S29*

1. NMR spectra

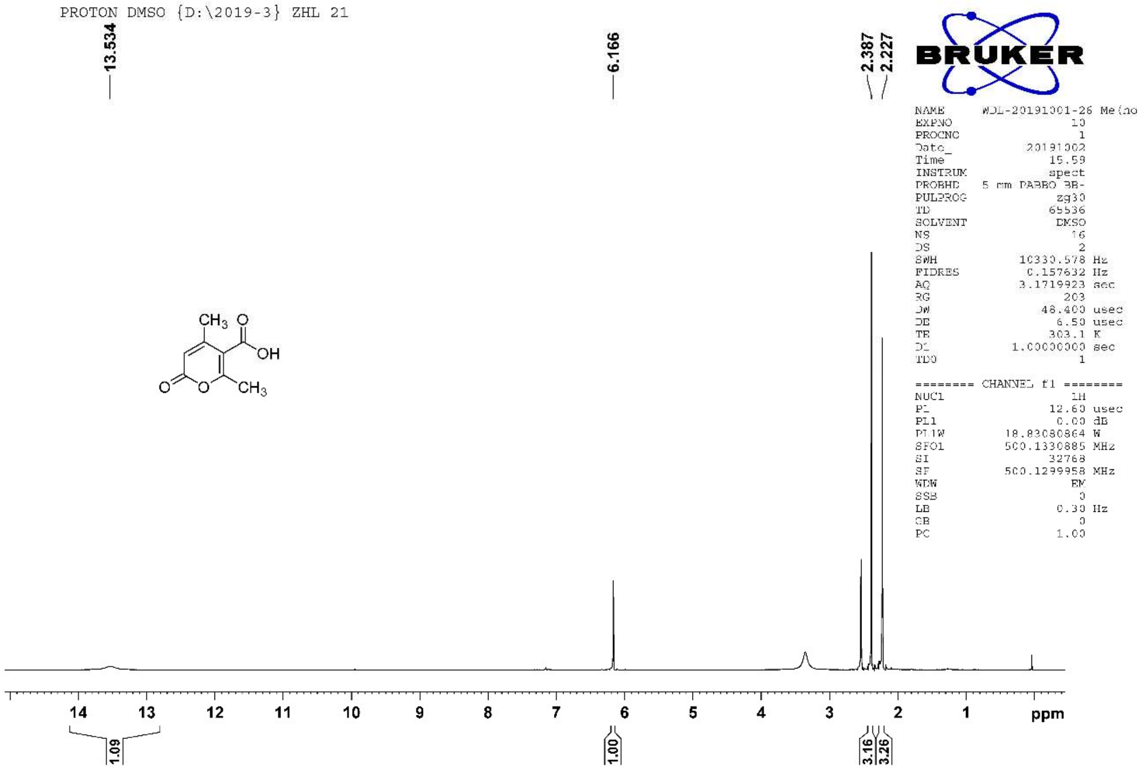

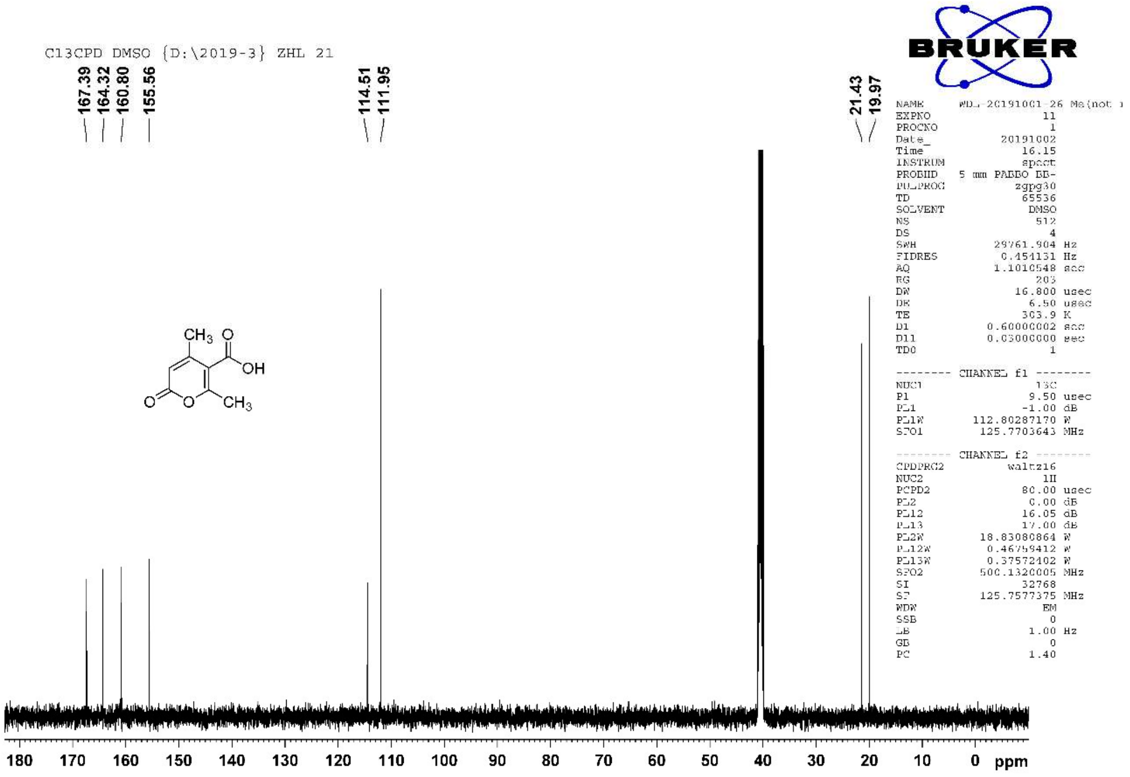

^1H and ^{13}C-NMR spectra of isodehydracetic acid.

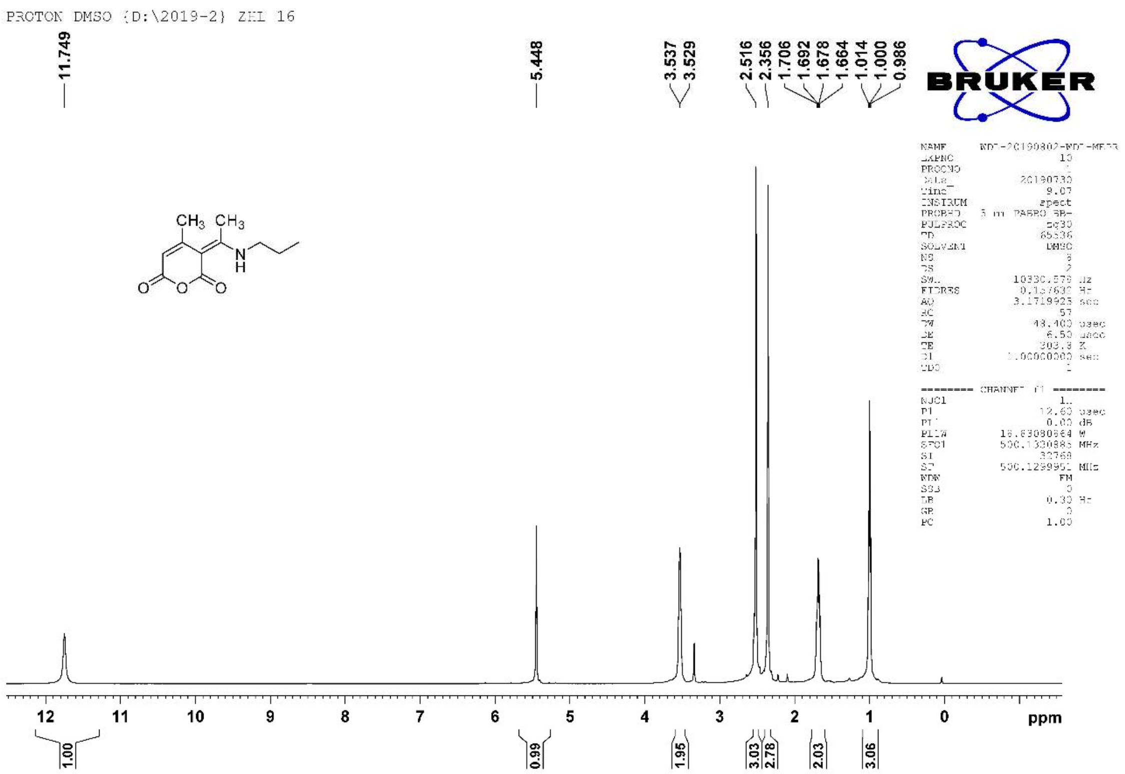

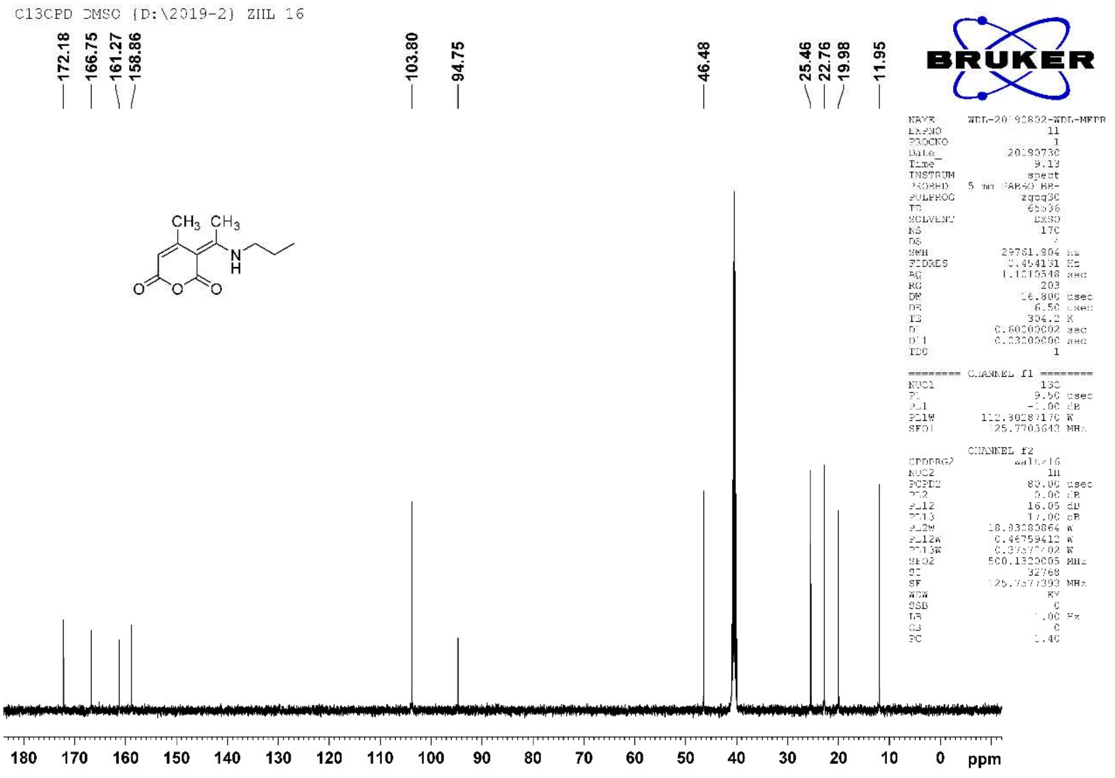

^{1}H and ^{13}C-NMR spectra of compound 2.

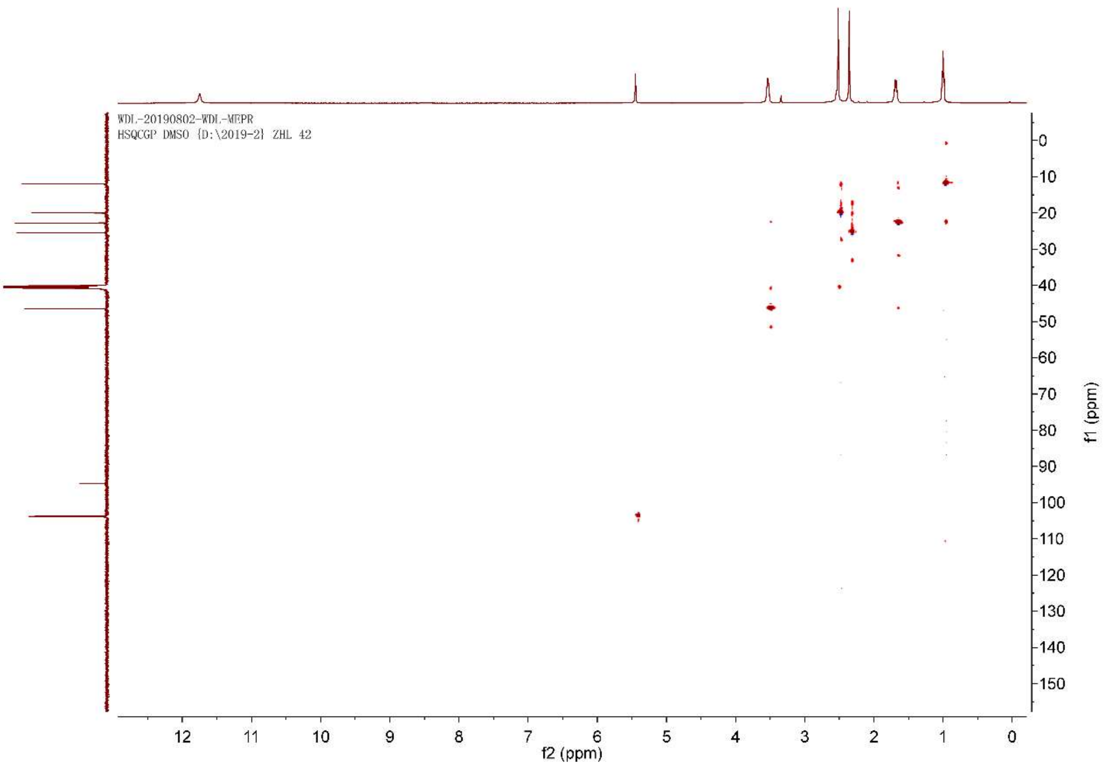

HSQC spectrum of compound 1.

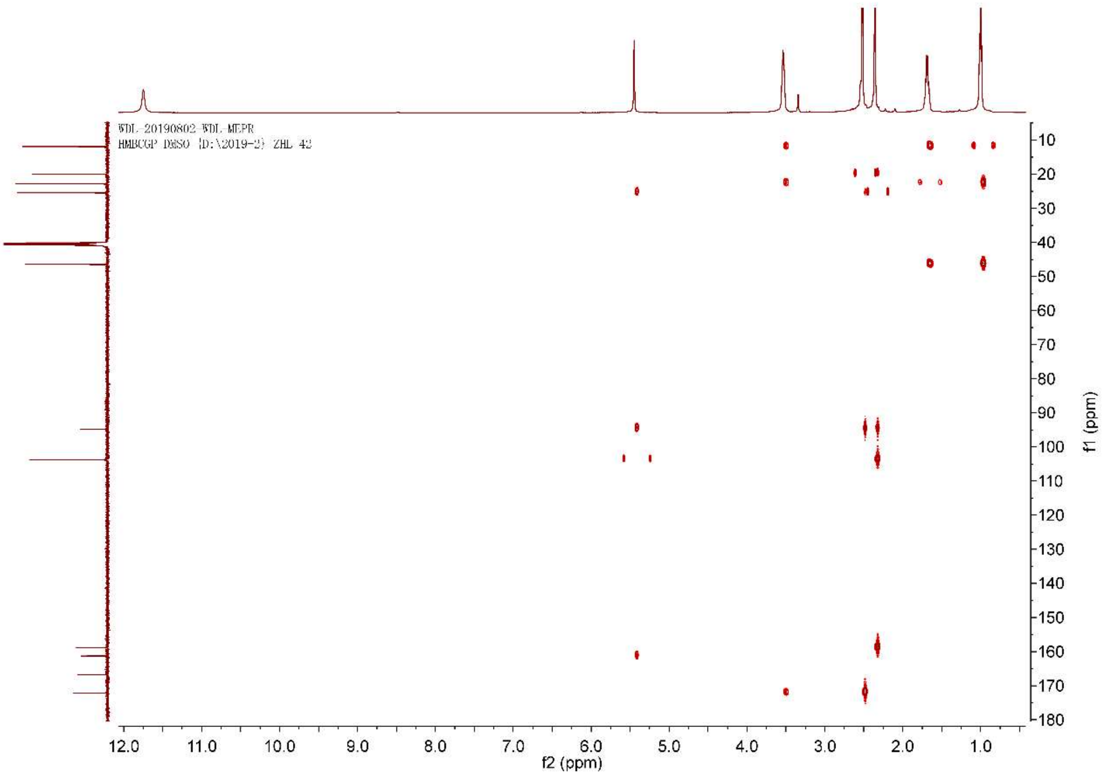

HMBC spectrum of compound 2.

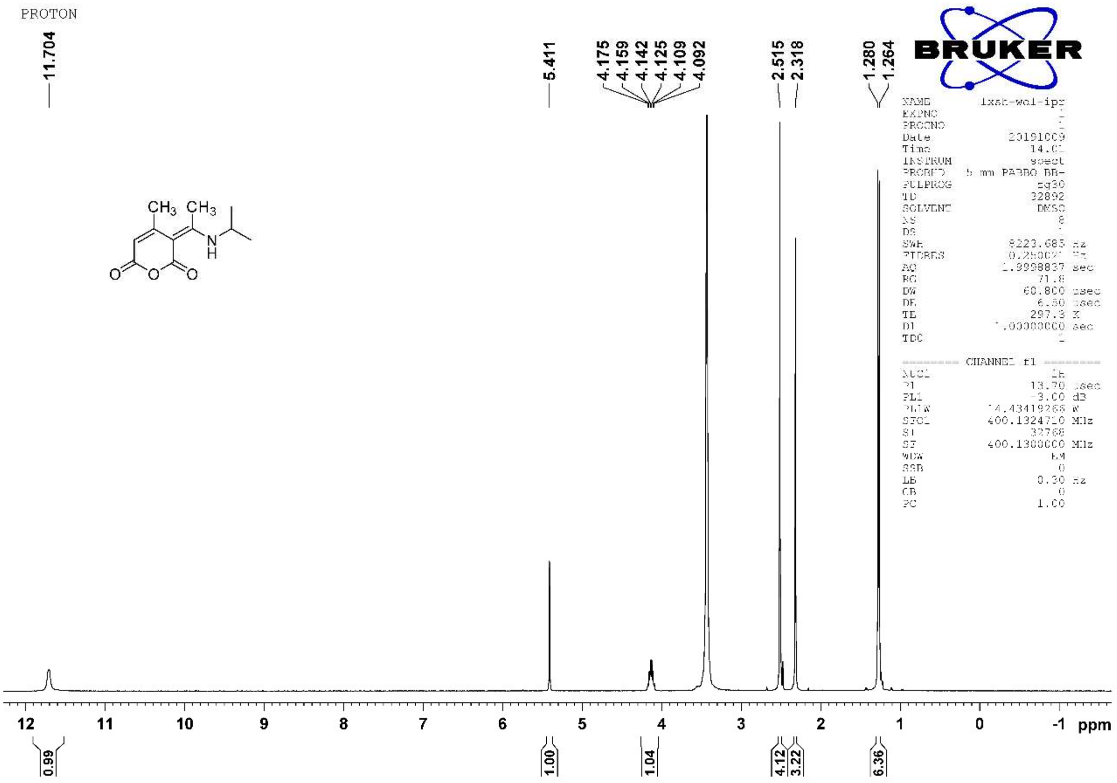

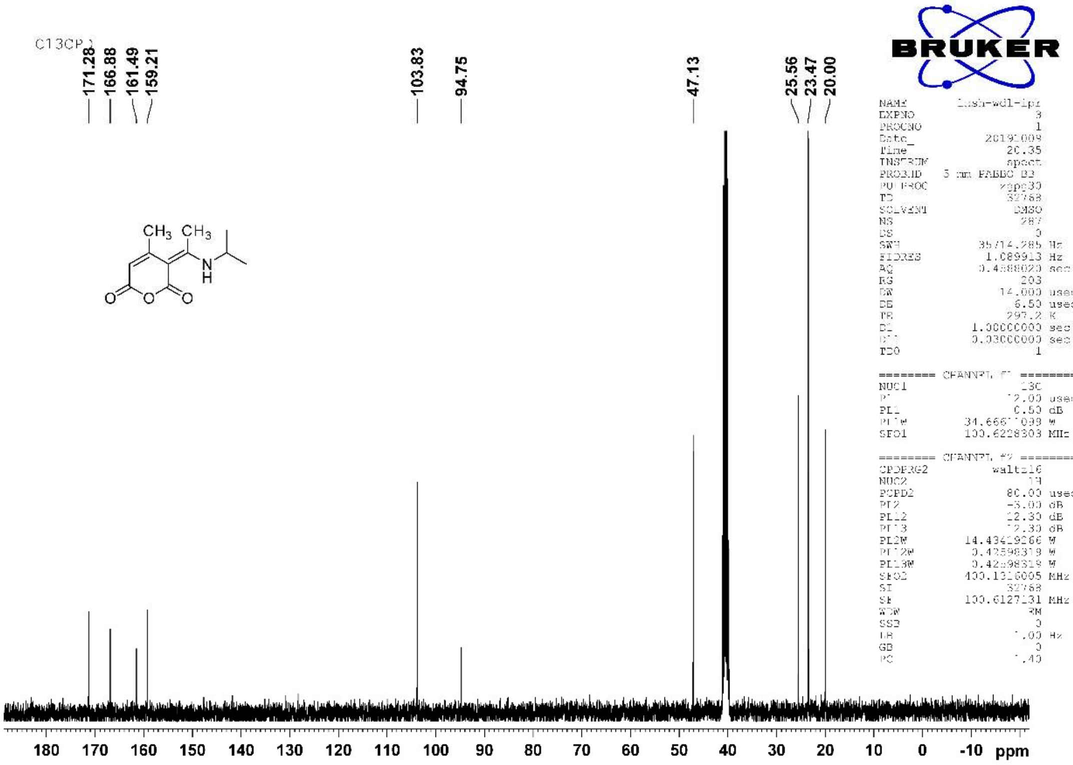

^{1}H and ^{13}C-NMR spectra of compound 4a.

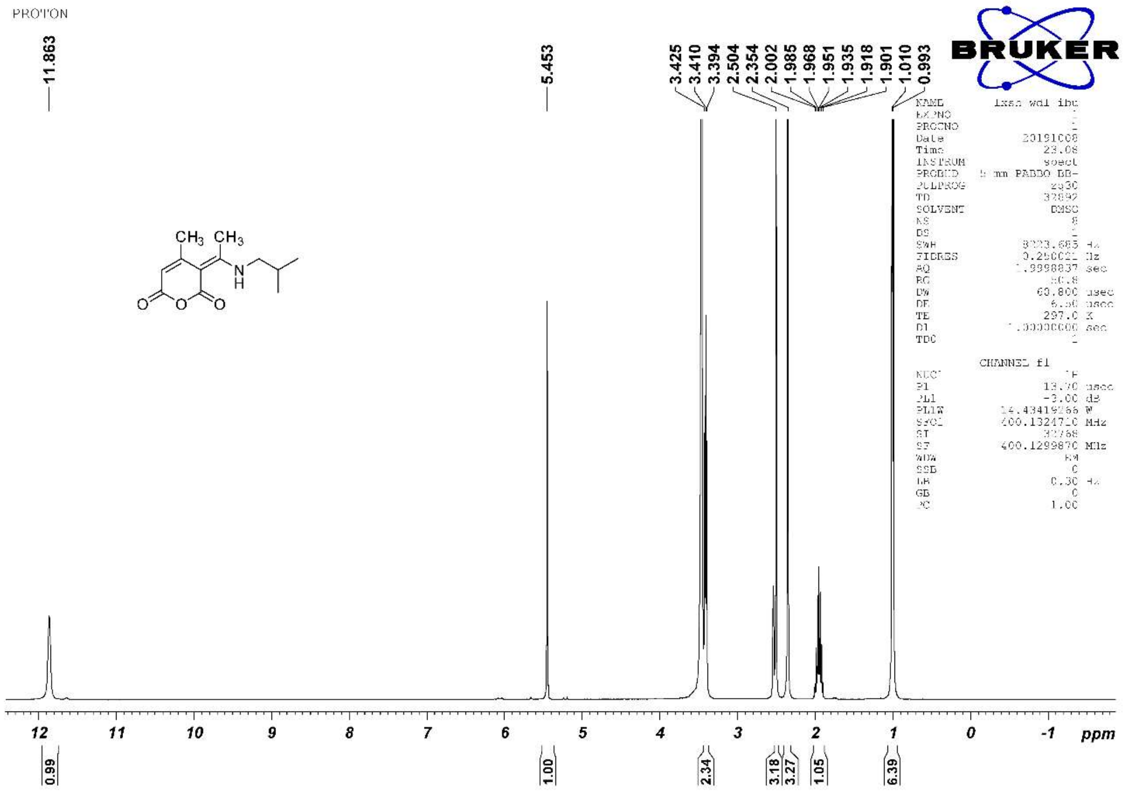

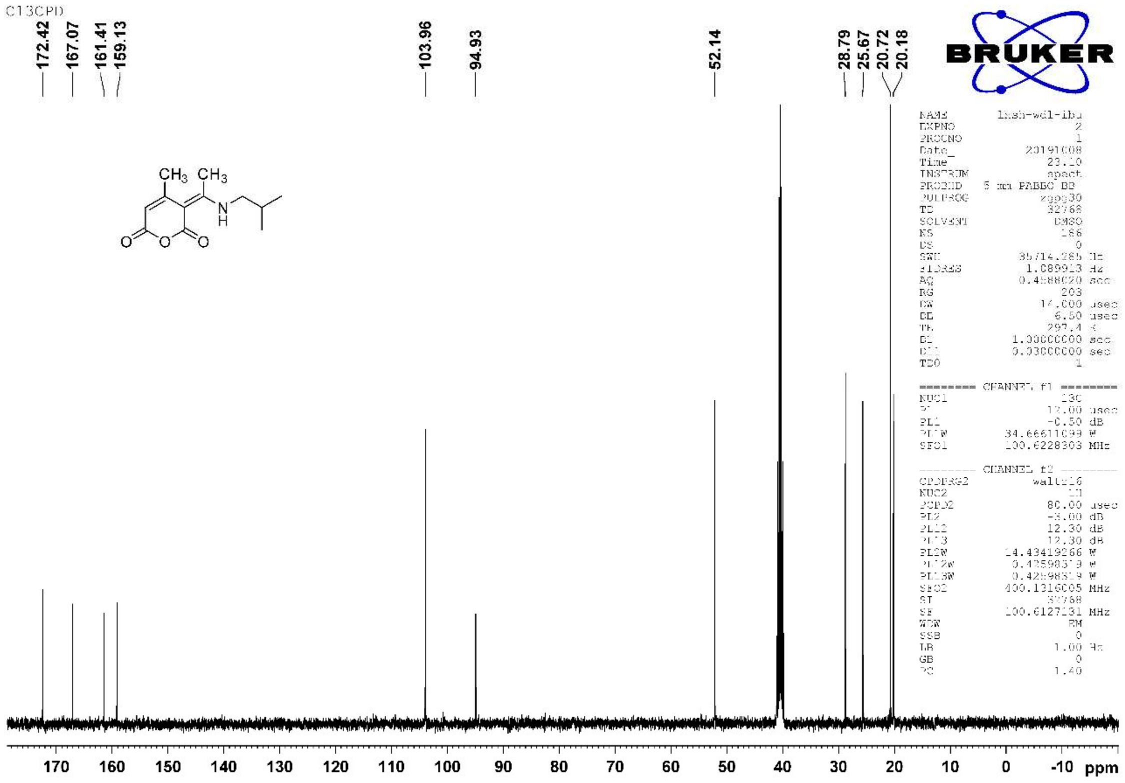

^{1}H and ^{13}C-NMR spectra of compound 3.

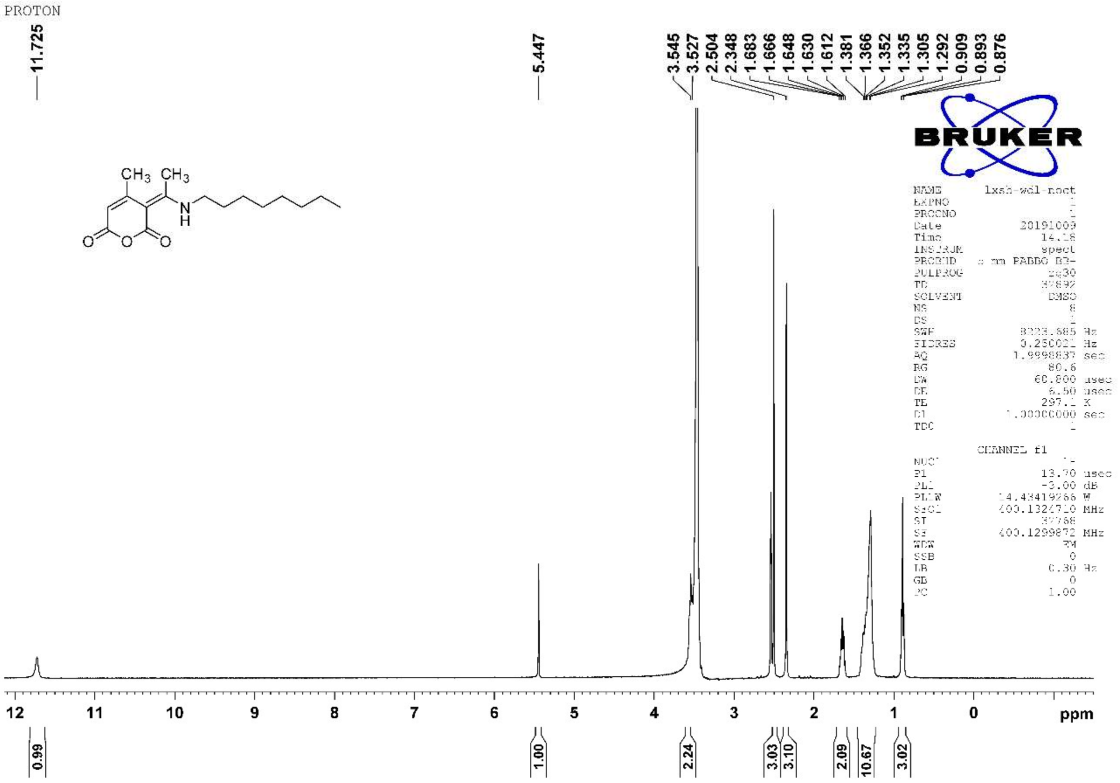

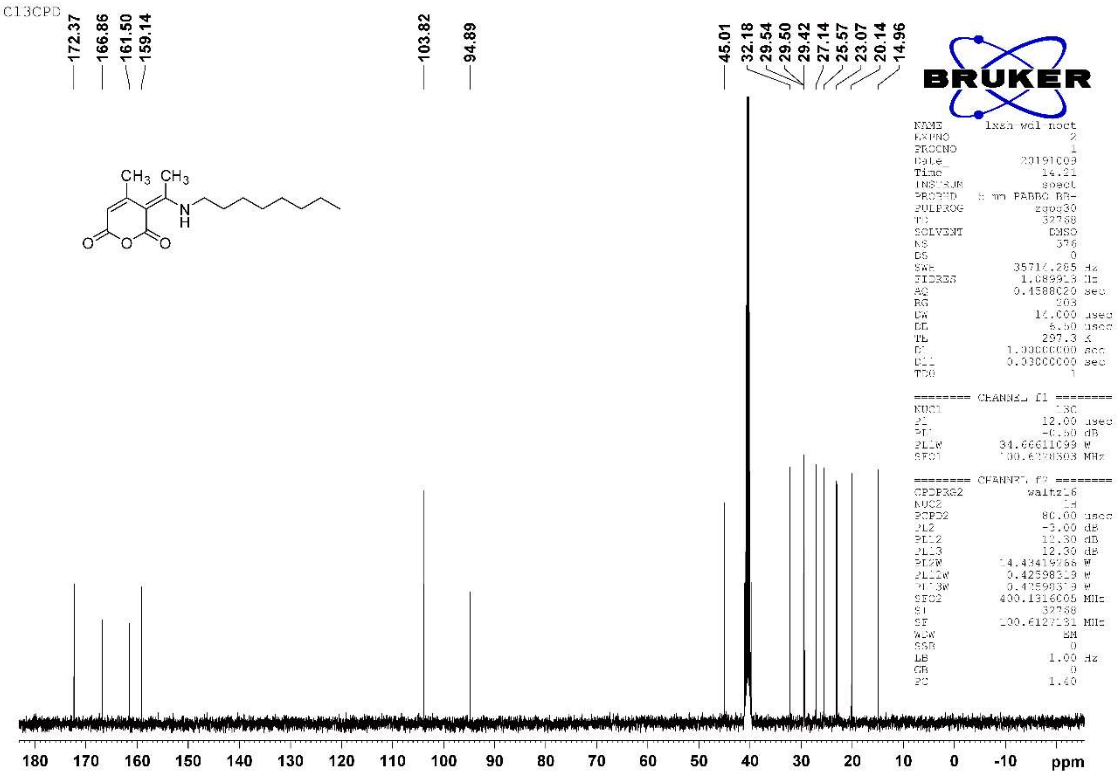

^{1}H and ^{13}C-NMR spectra of compound 4b.

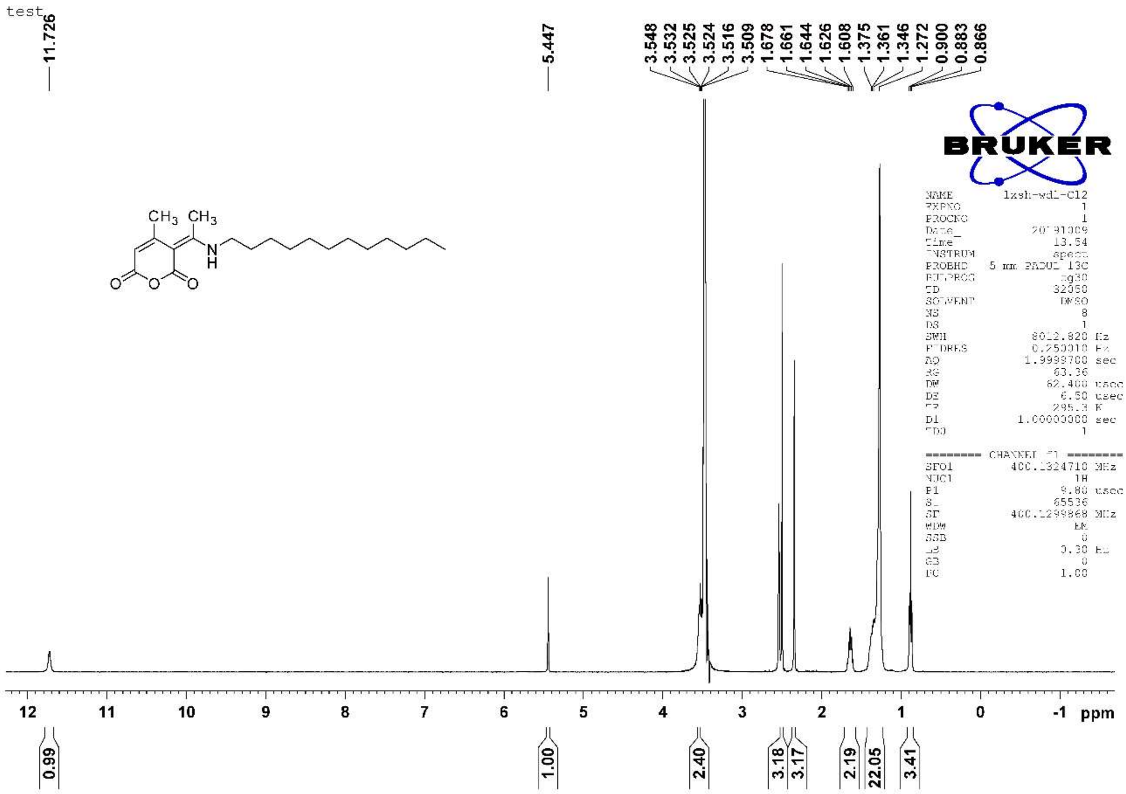

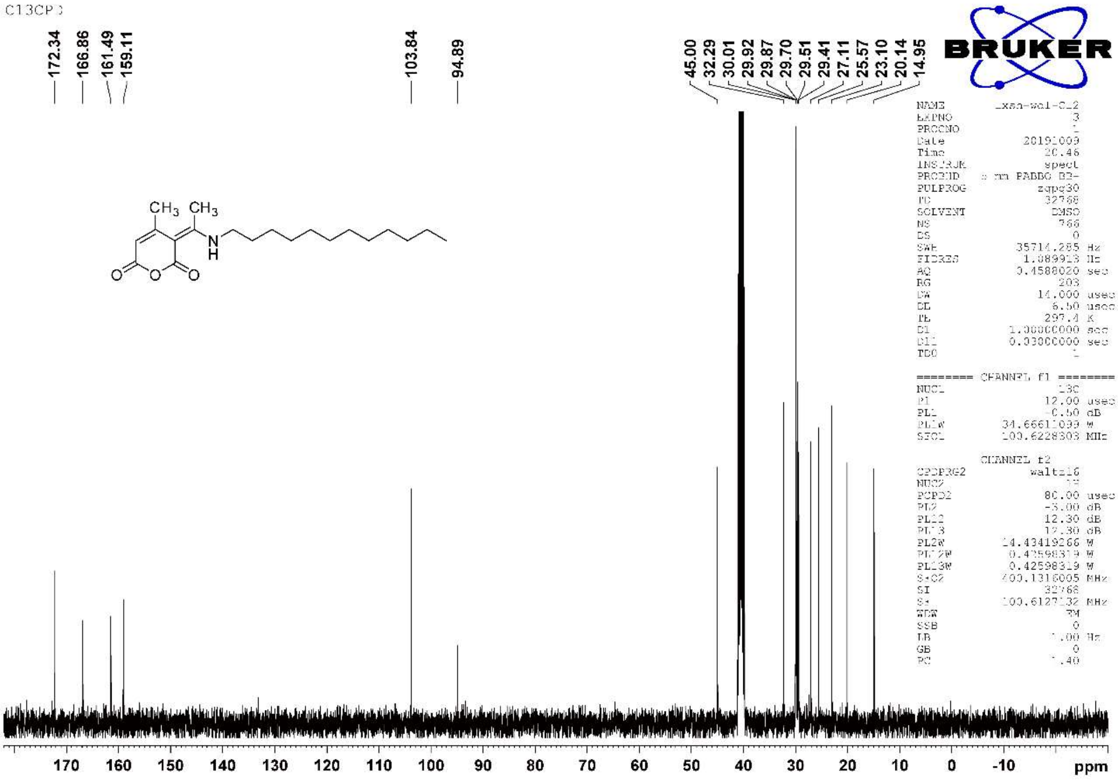

^{1}H and ^{13}C-NMR spectra of compound 4c.

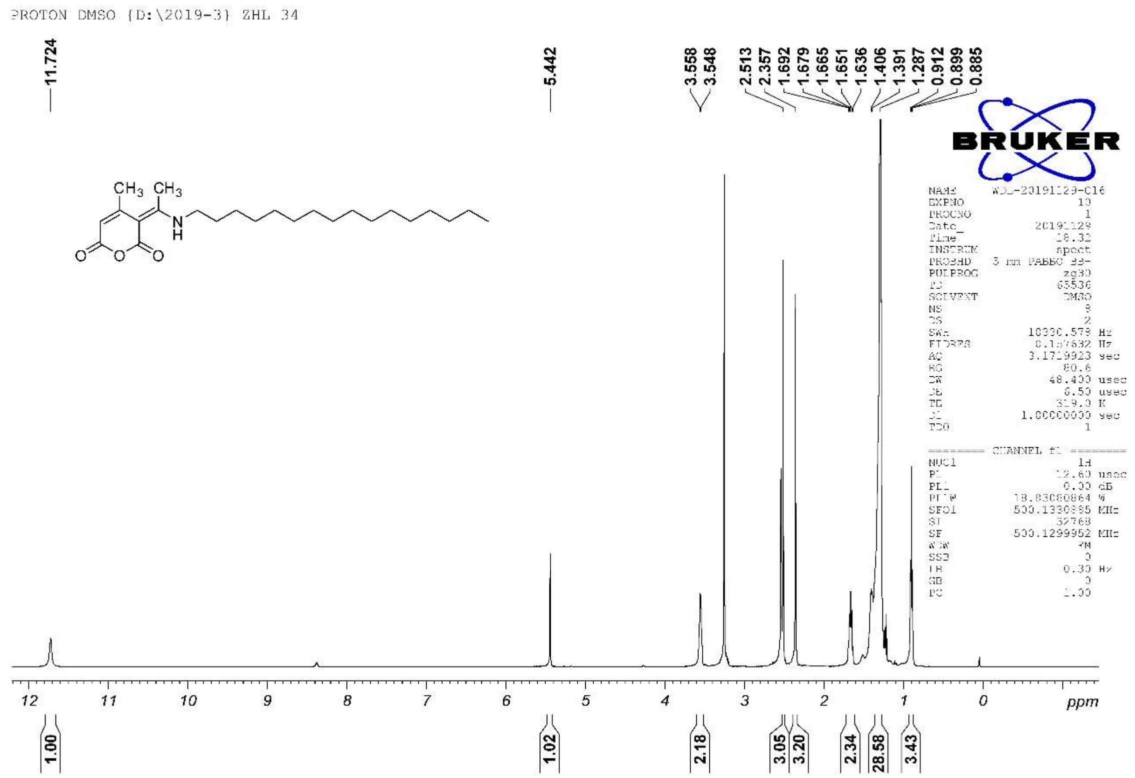

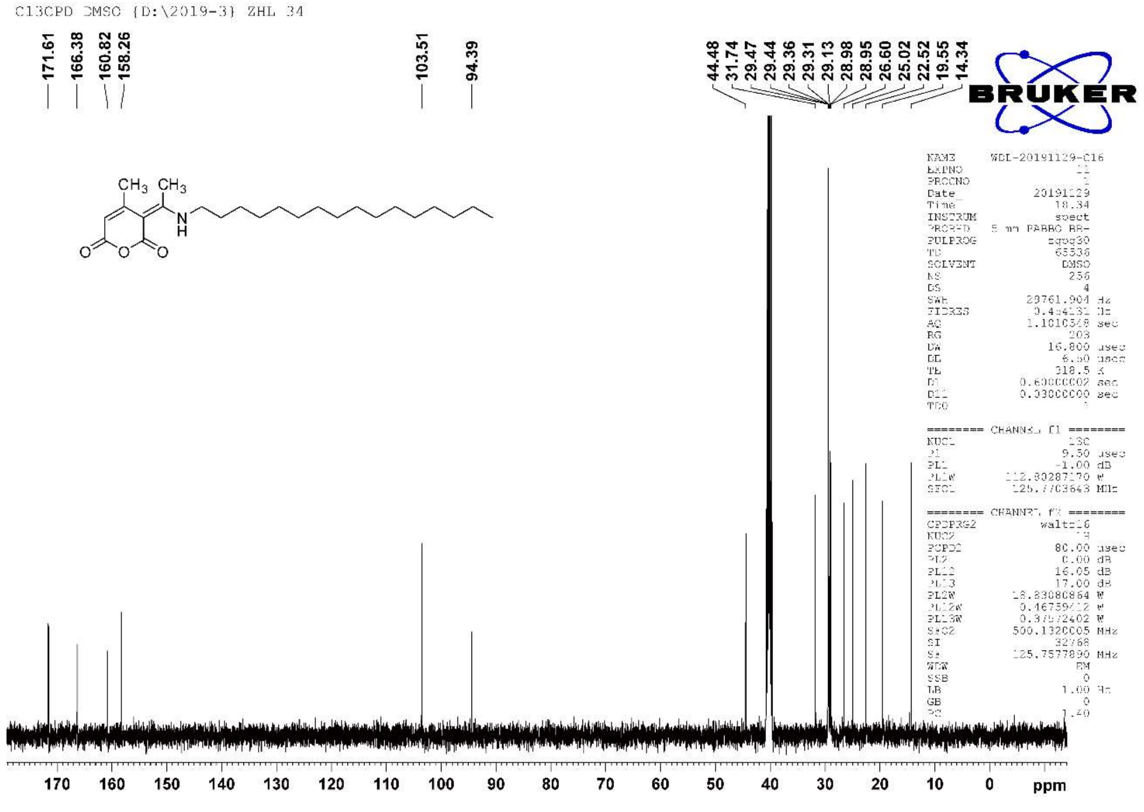

^{1}H and ^{13}C-NMR spectra of compound 4d.

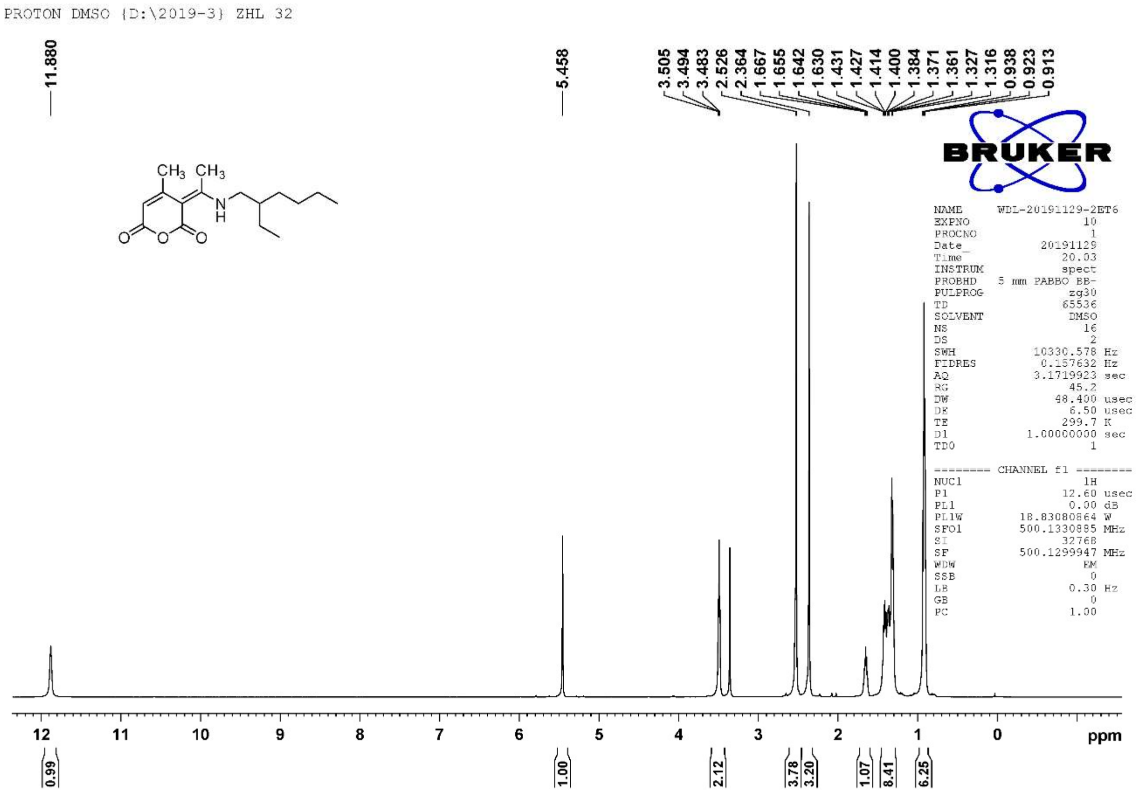

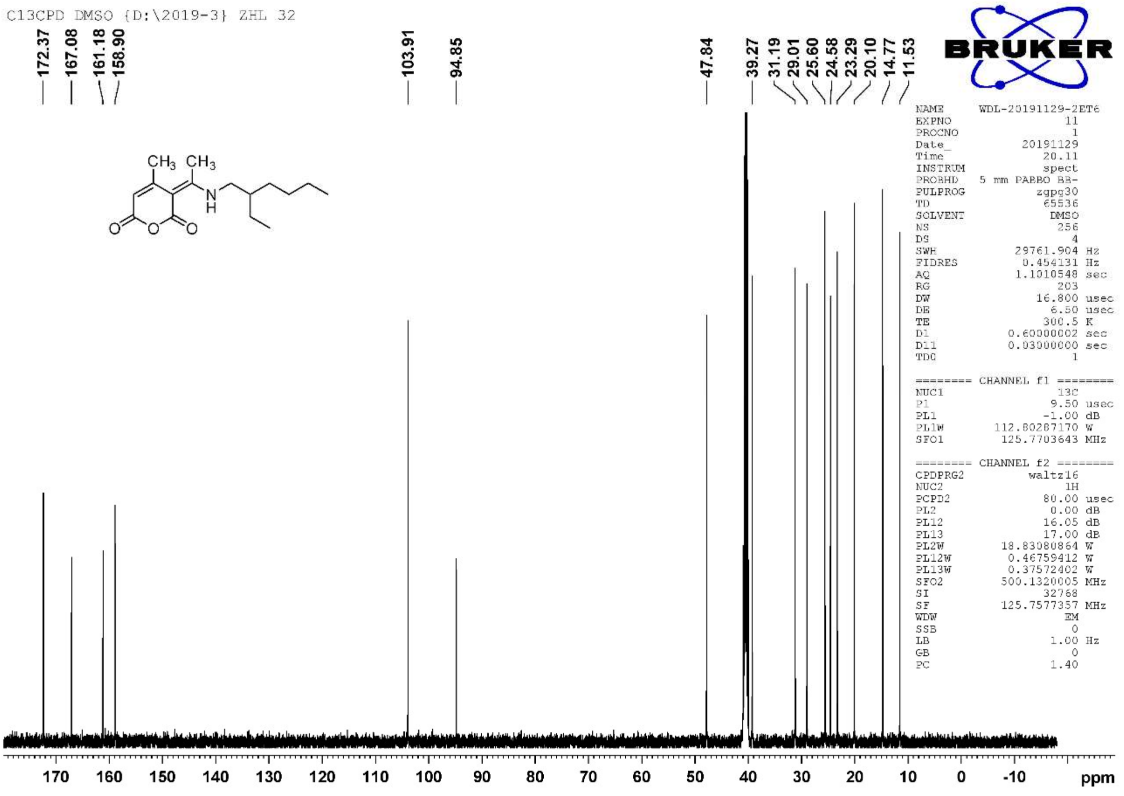

^{1}H and ^{13}C-NMR spectra of compound 4e.

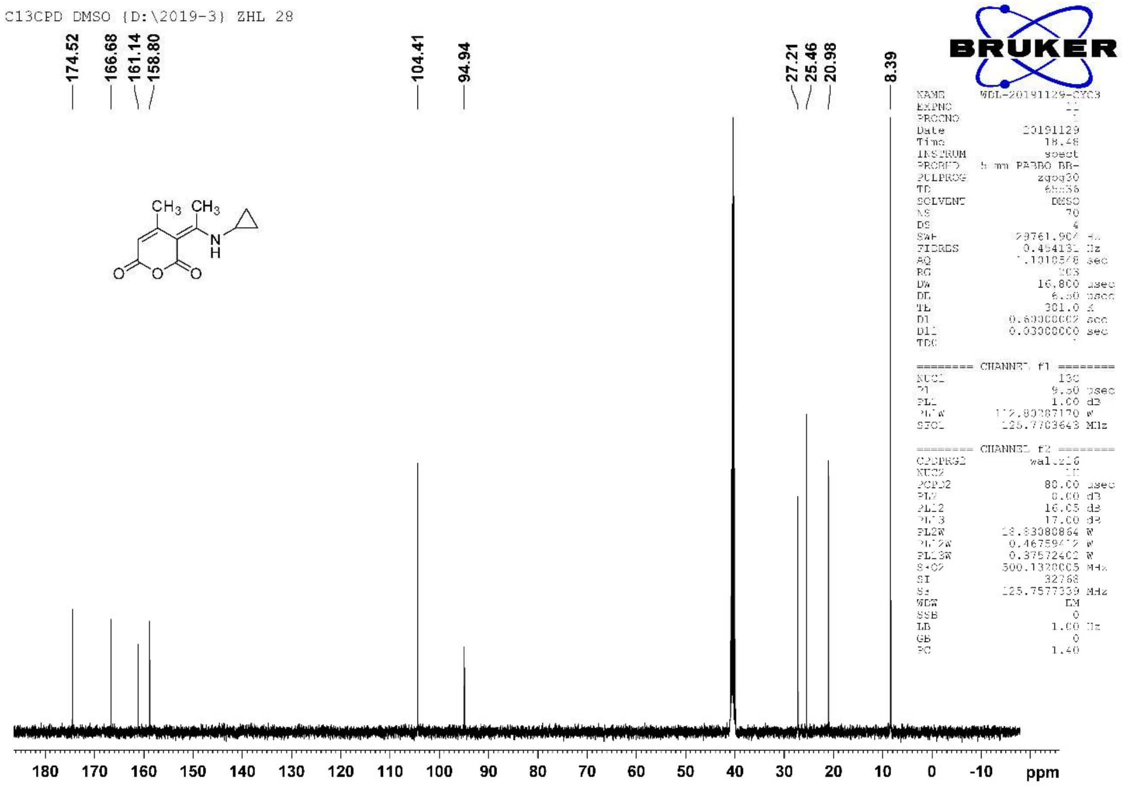

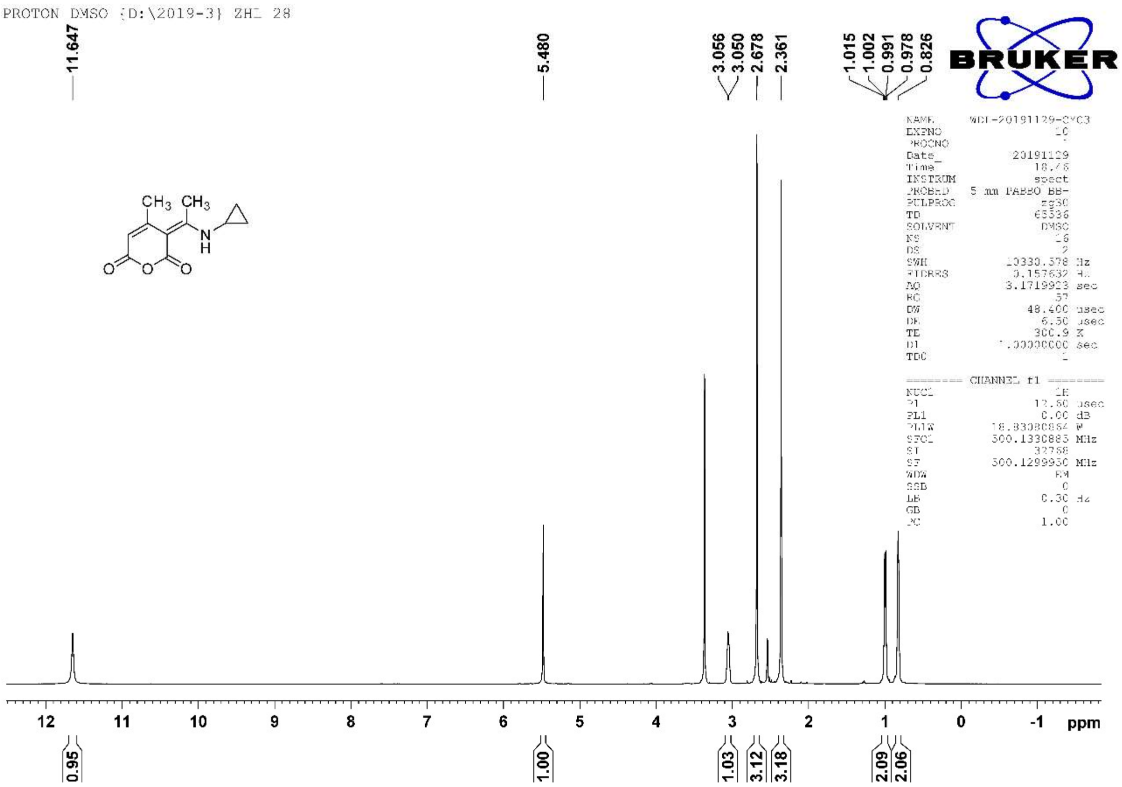

^{1}H and ^{13}C-NMR spectra of compound 4f.

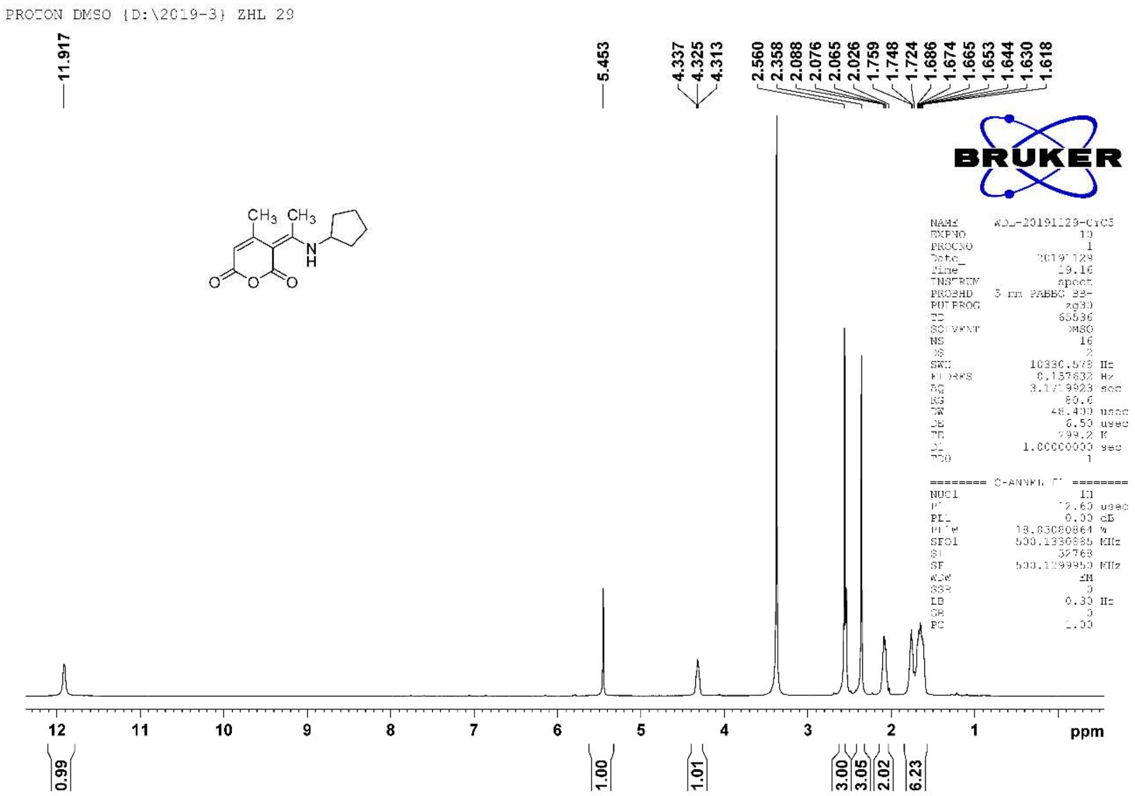

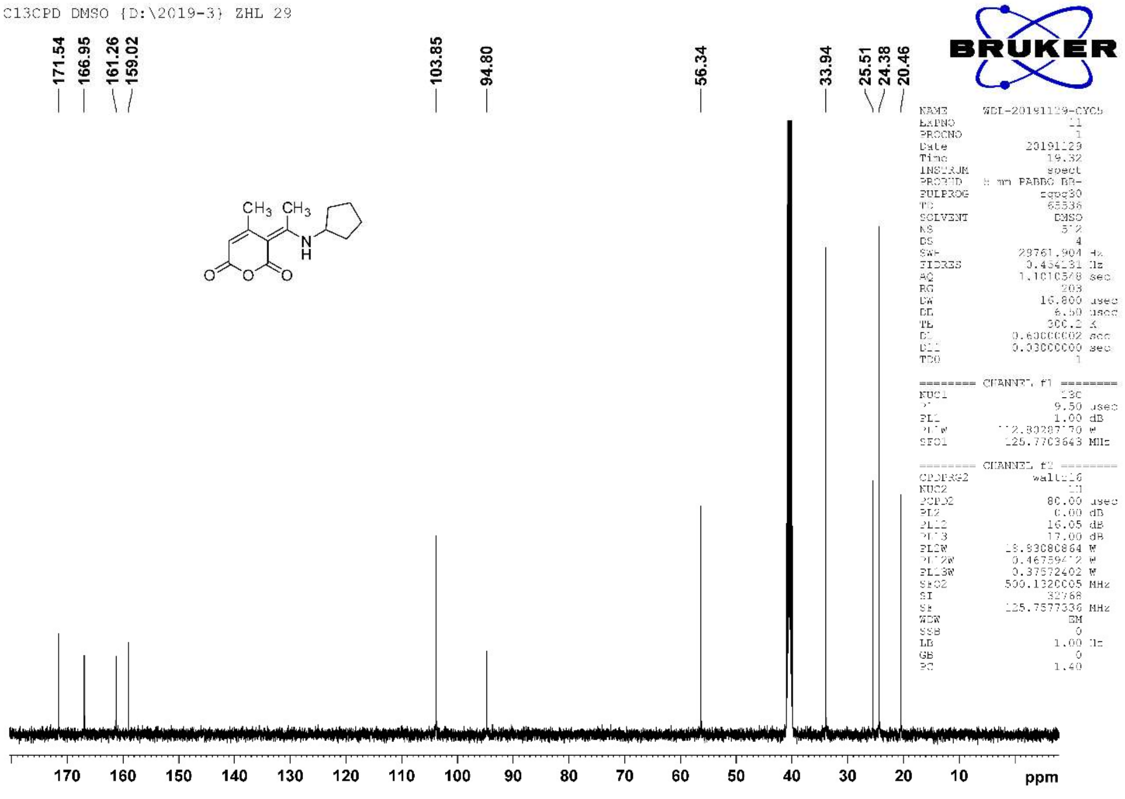

^{1}H and ^{13}C-NMR spectra of compound 4g.

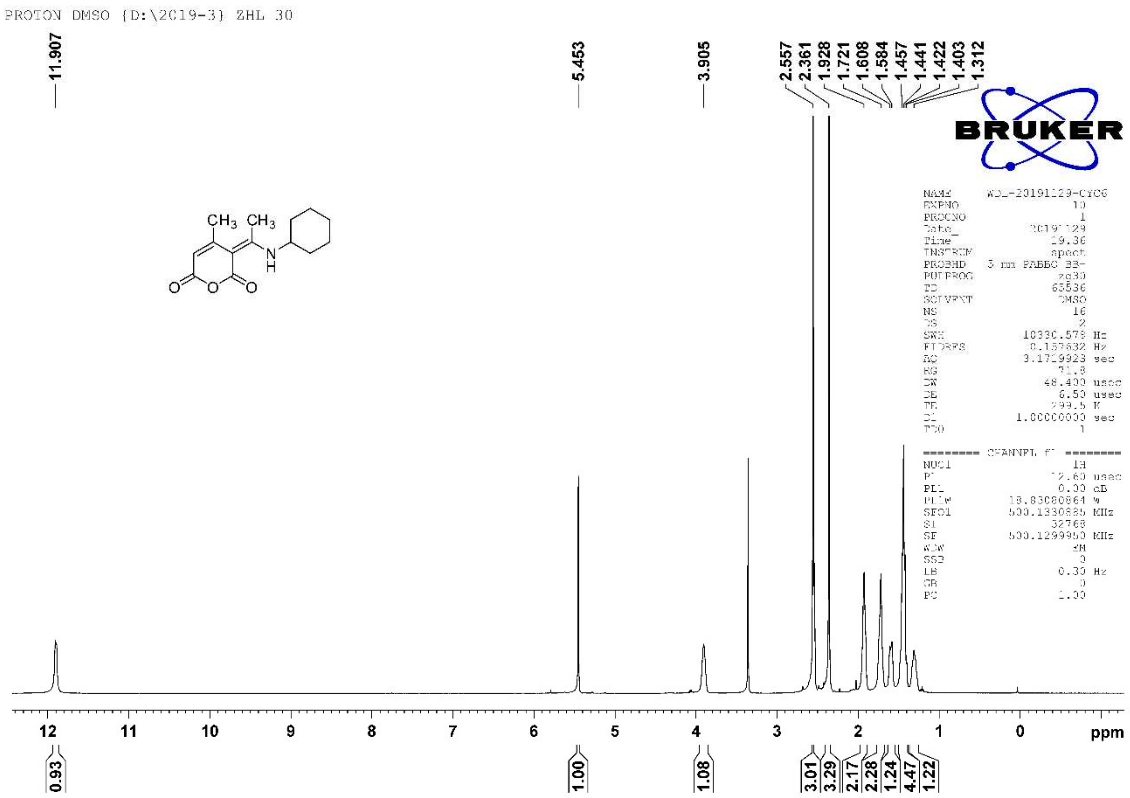

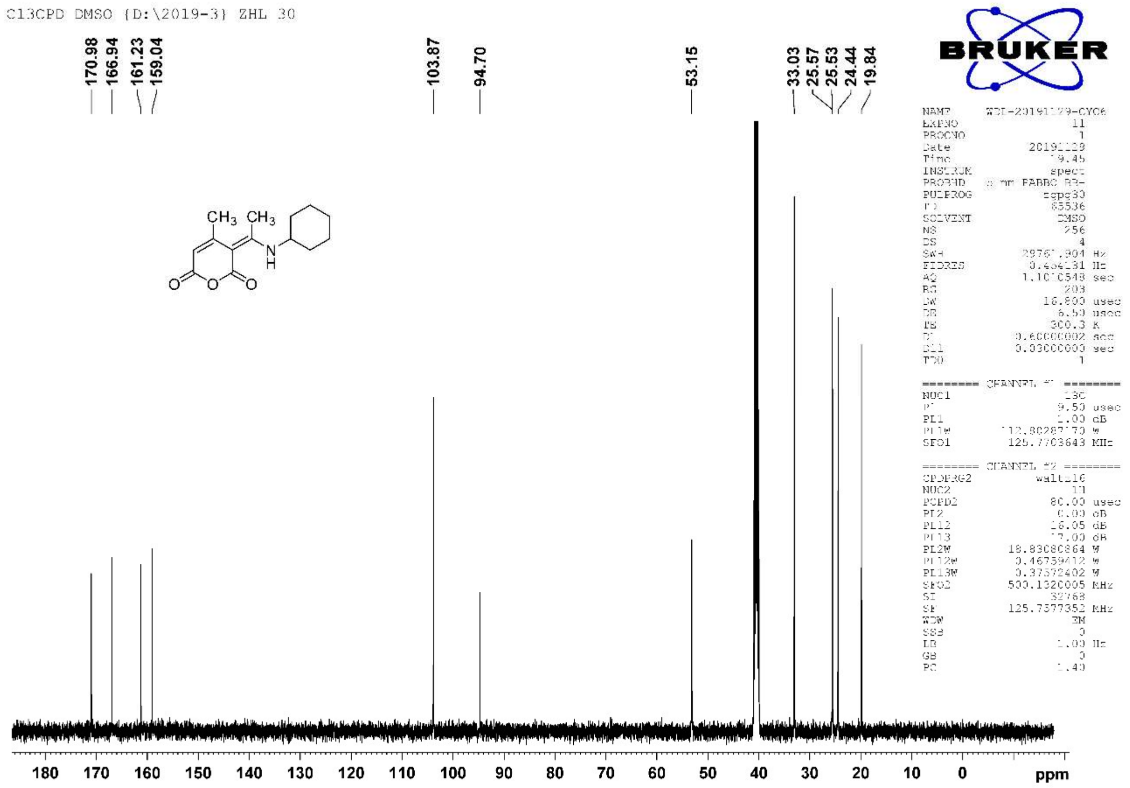

^{1}H and ^{13}C-NMR spectra of compound 4h.

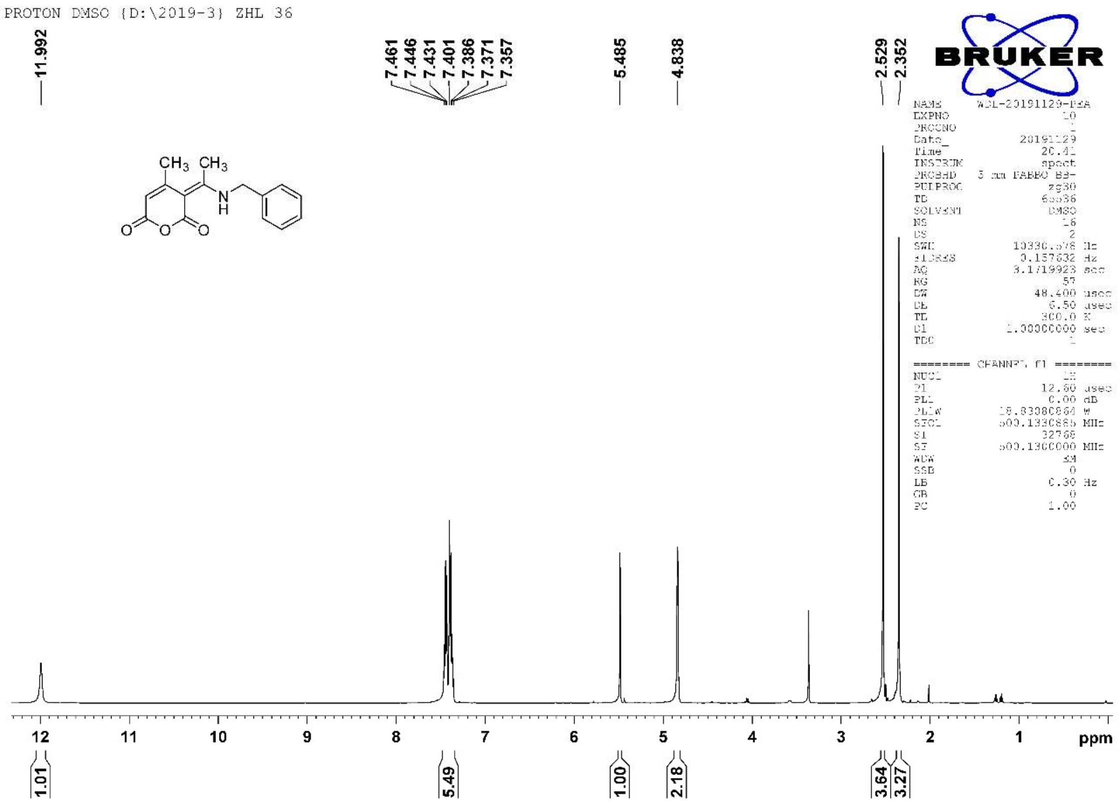

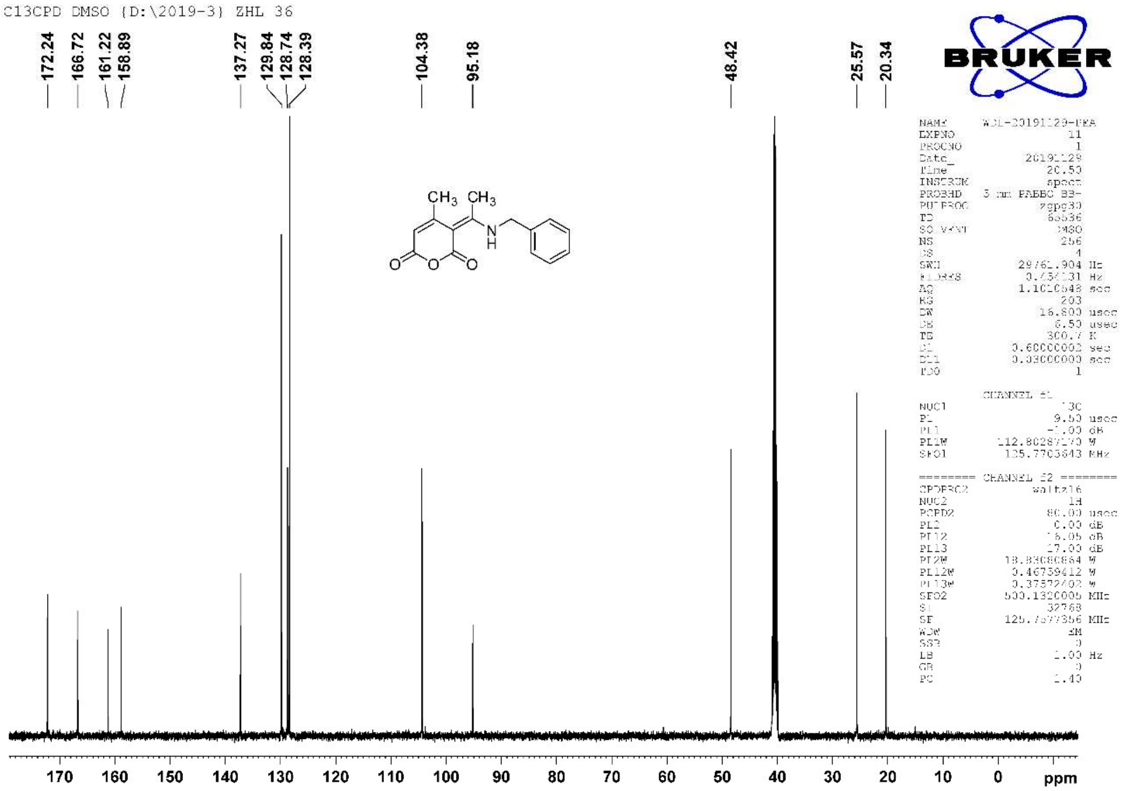

^{1}H and ^{13}C-NMR spectra of compound 4i.

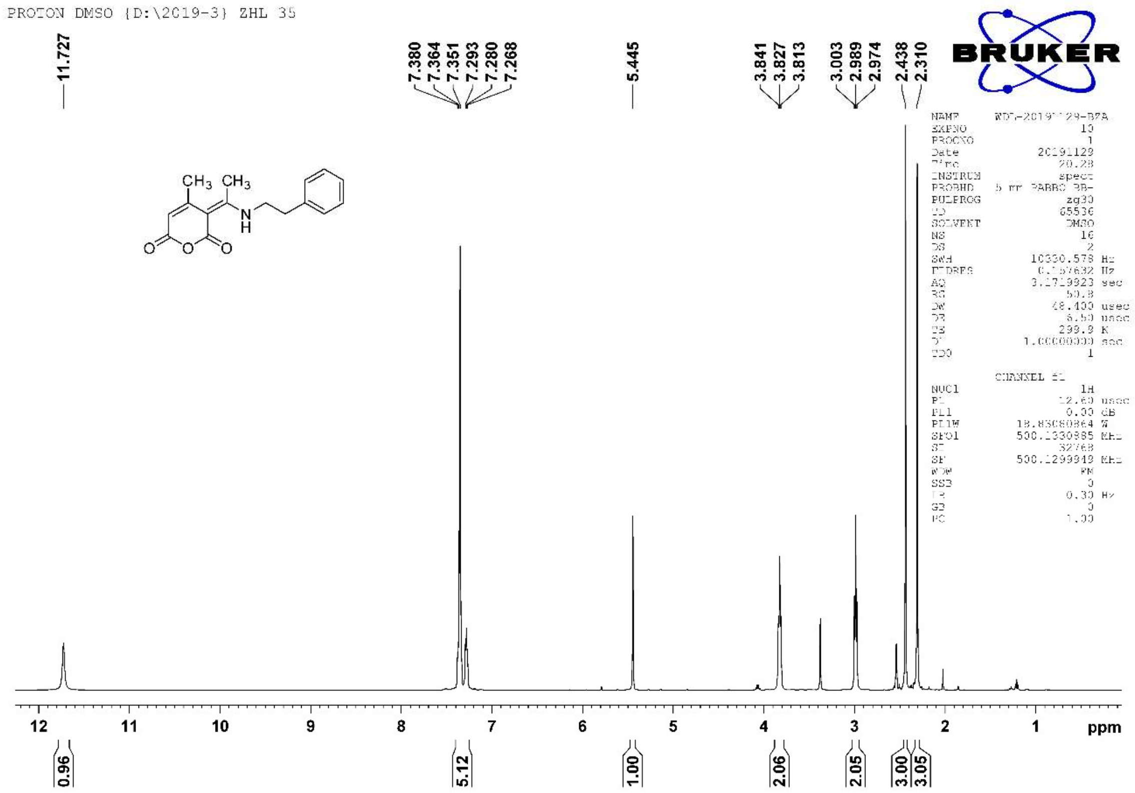

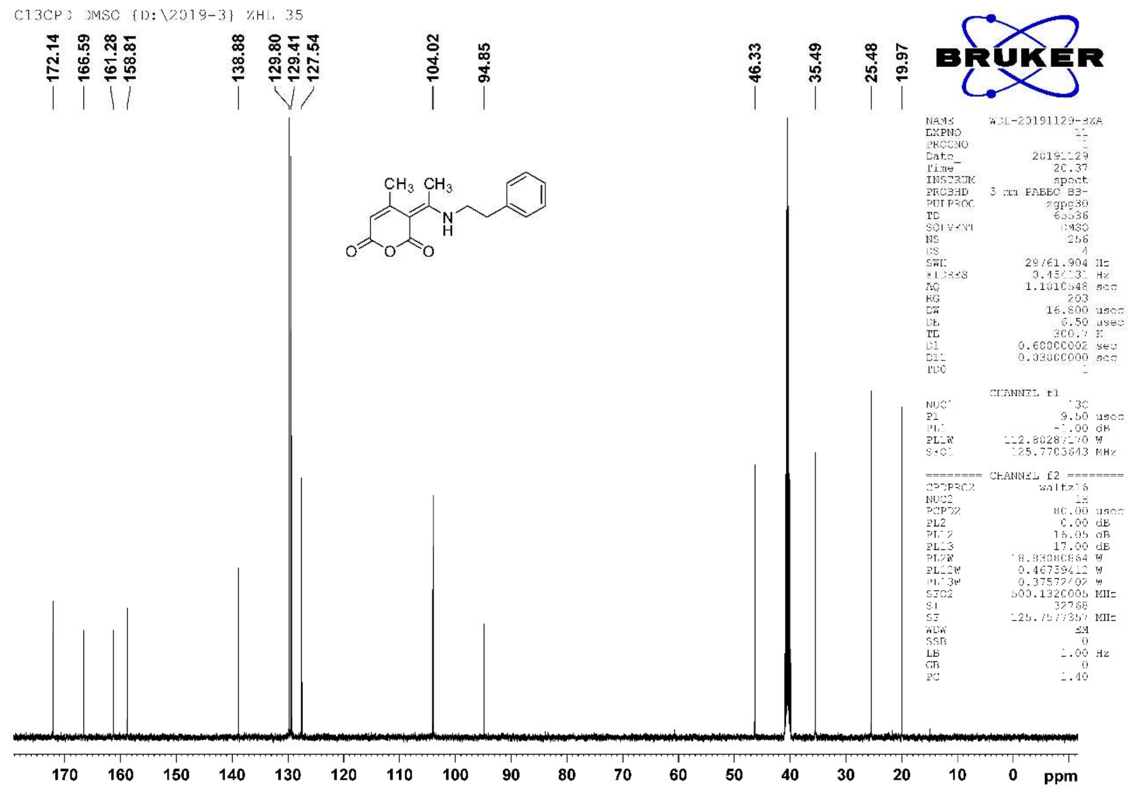

^{1}H and ^{13}C-NMR spectra of compound 4j.

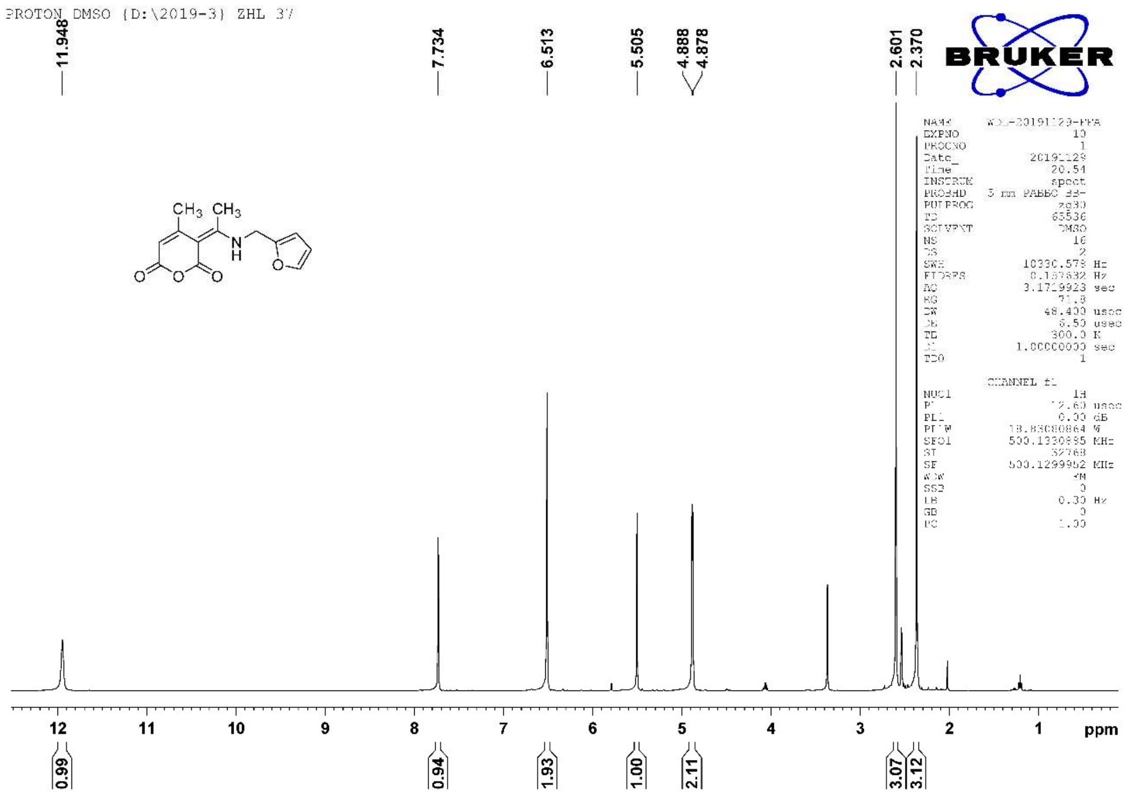

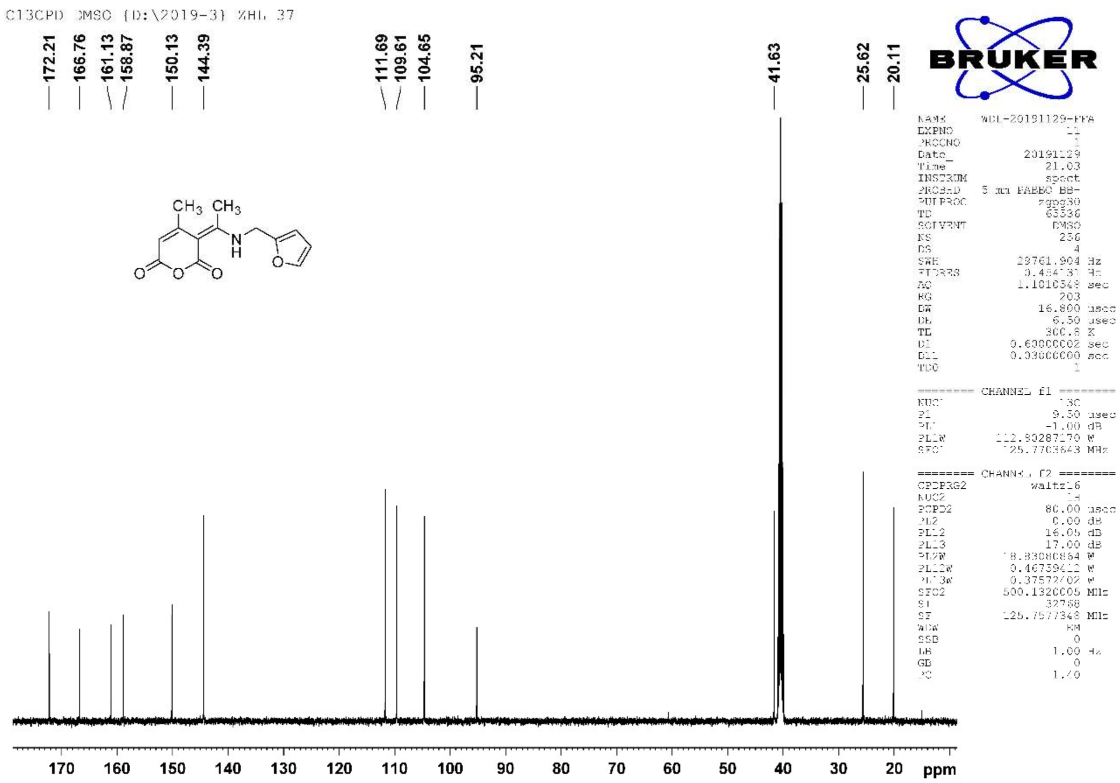

^{1}H and ^{13}C-NMR spectra of compound 4k.

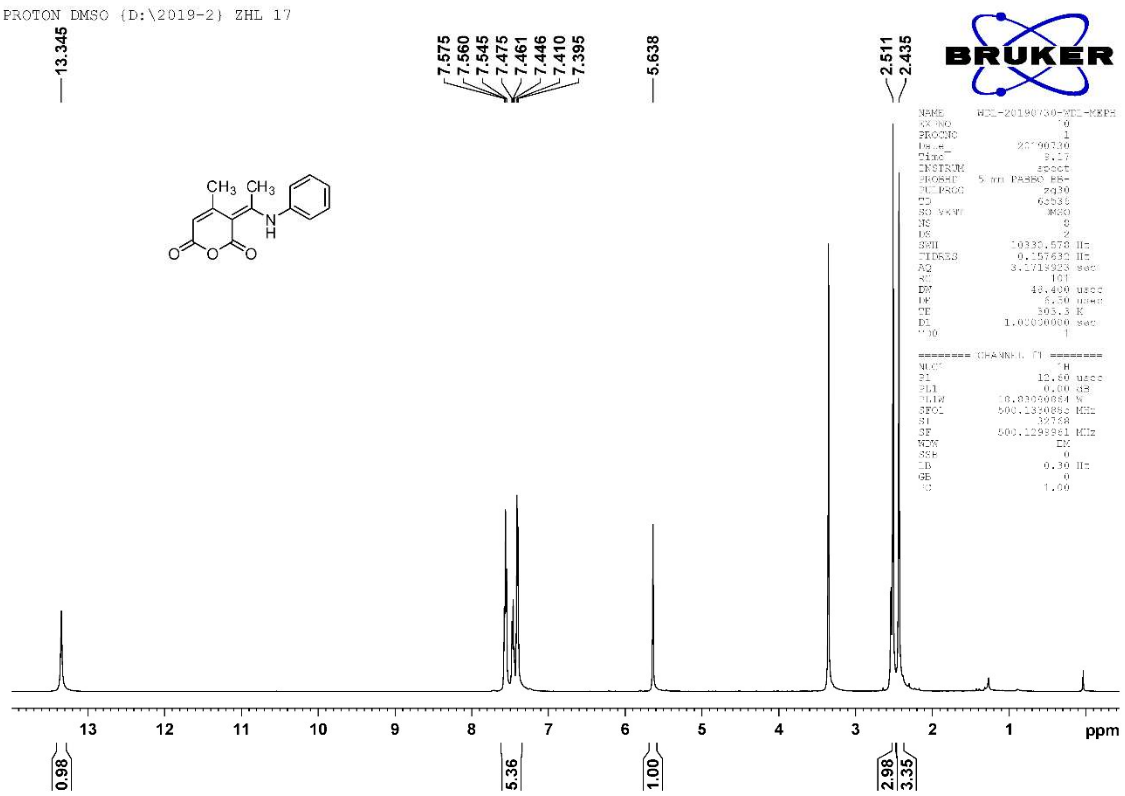

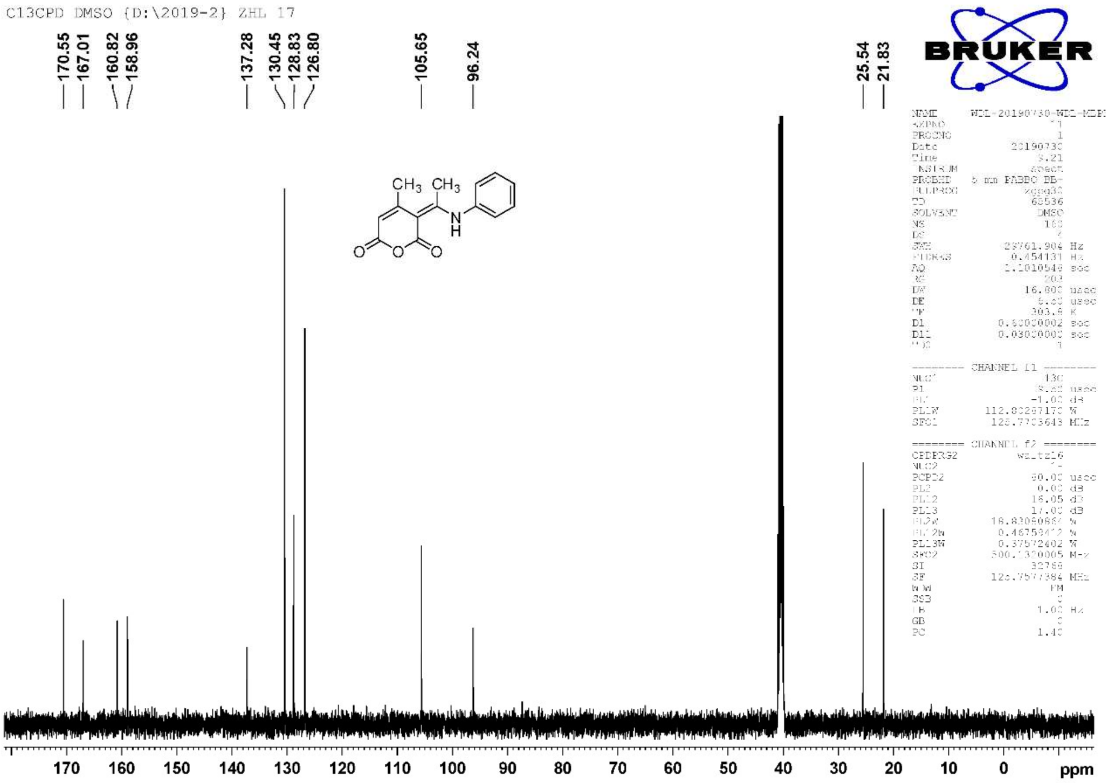

^{1}H and ^{13}C-NMR spectra of compound 4l.

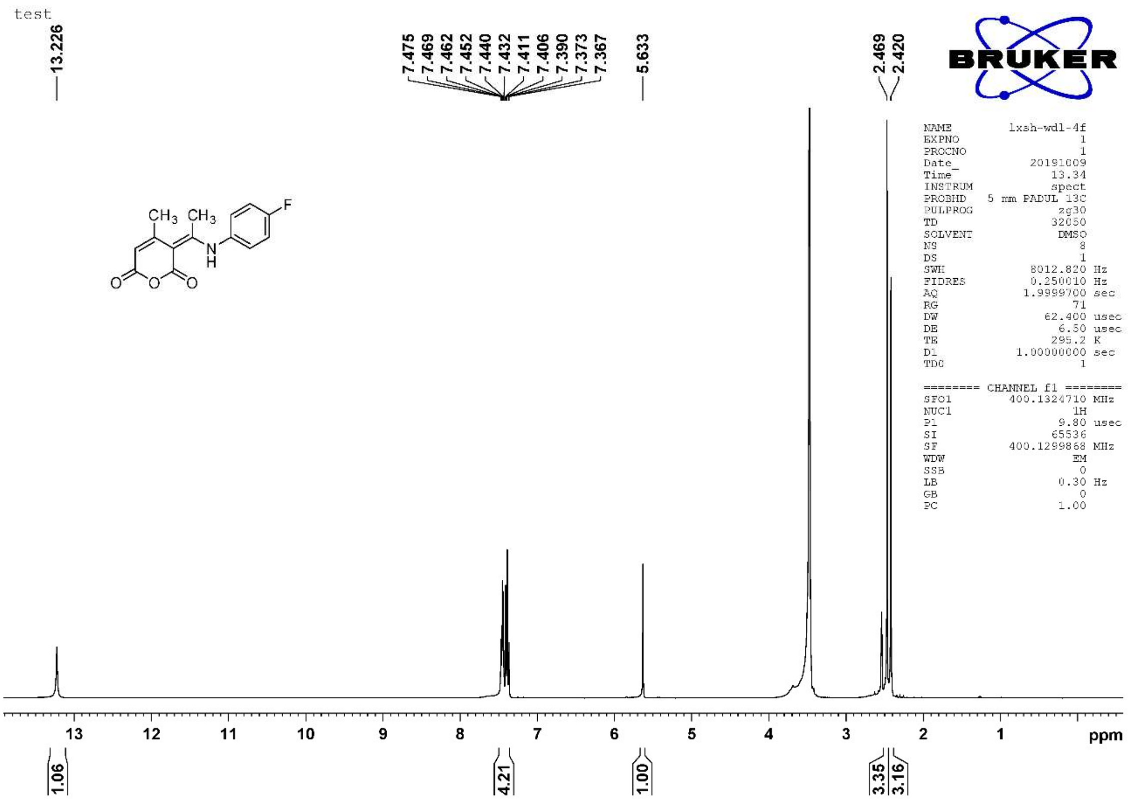

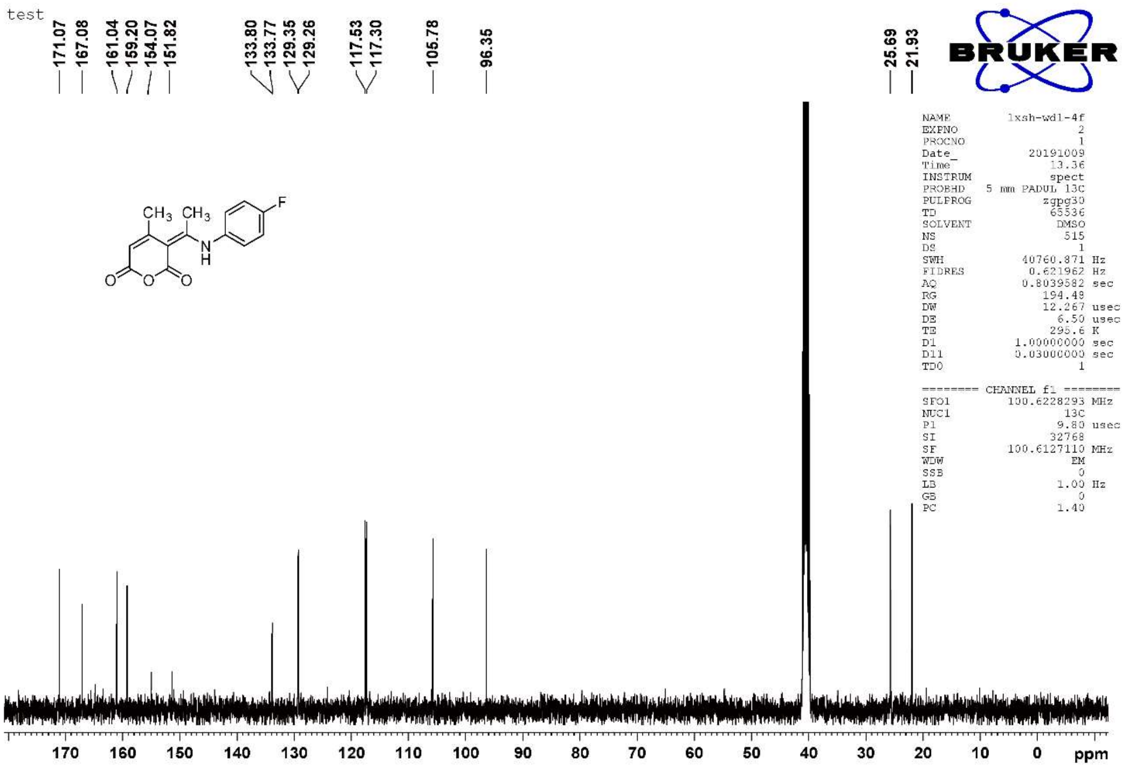

^{1}H and ^{13}C-NMR spectra of compound 4m.

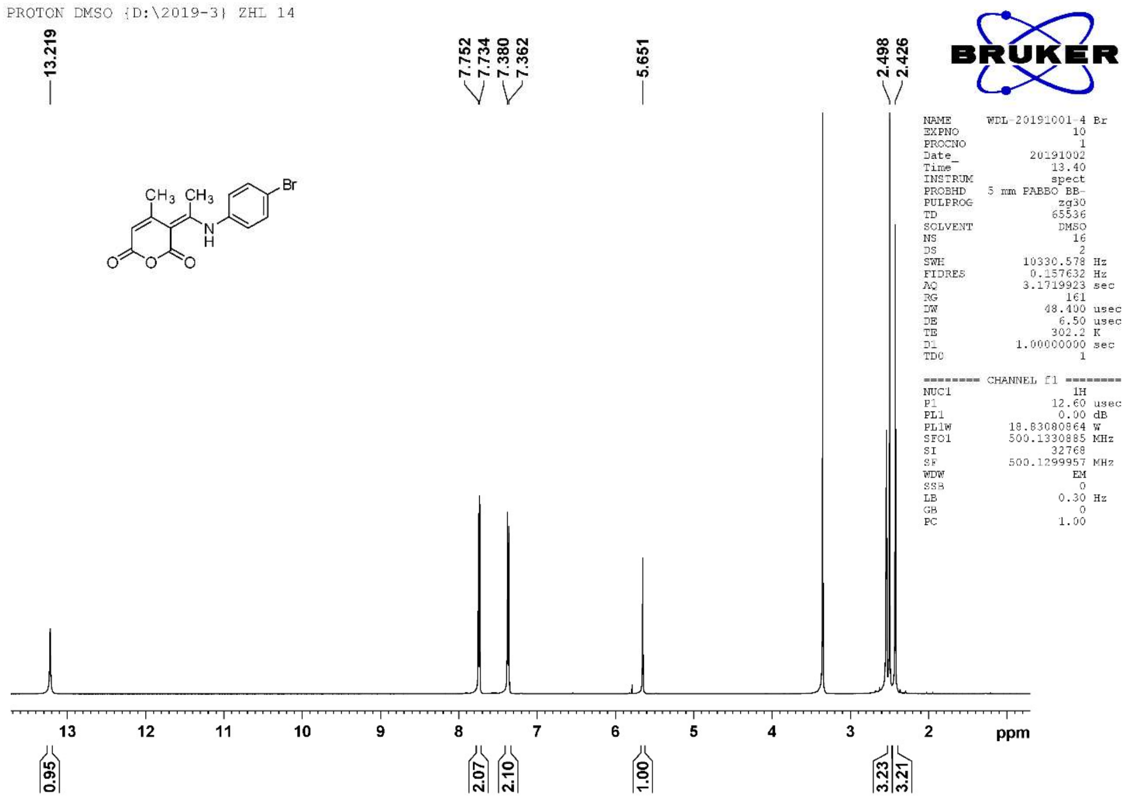

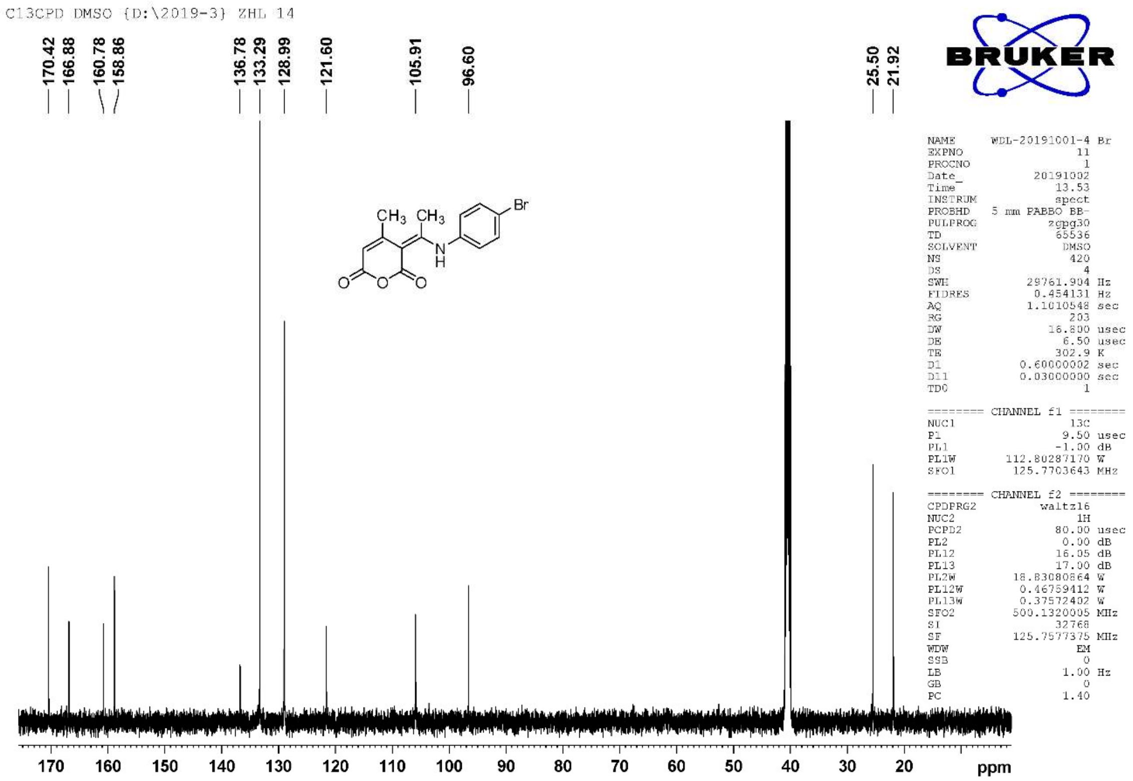

^{1}H and ^{13}C-NMR spectra of compound 4n.

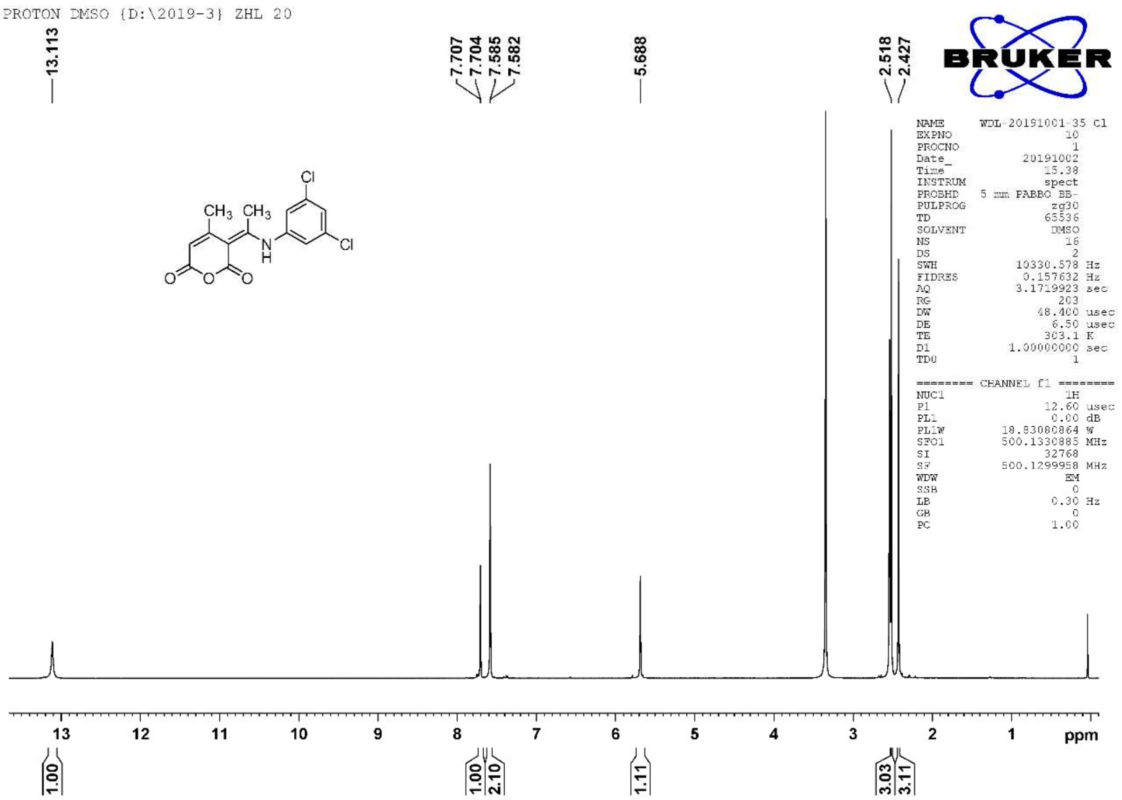

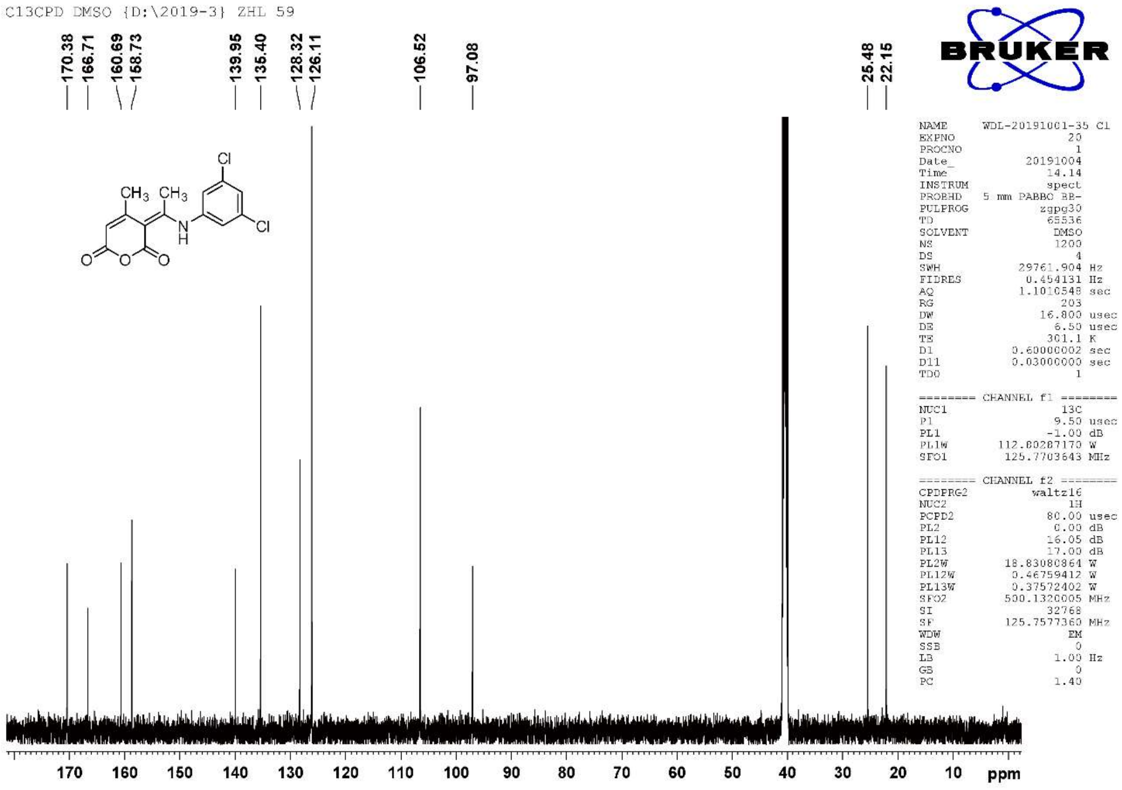

^{1}H and ^{13}C-NMR spectra of compound 4o.

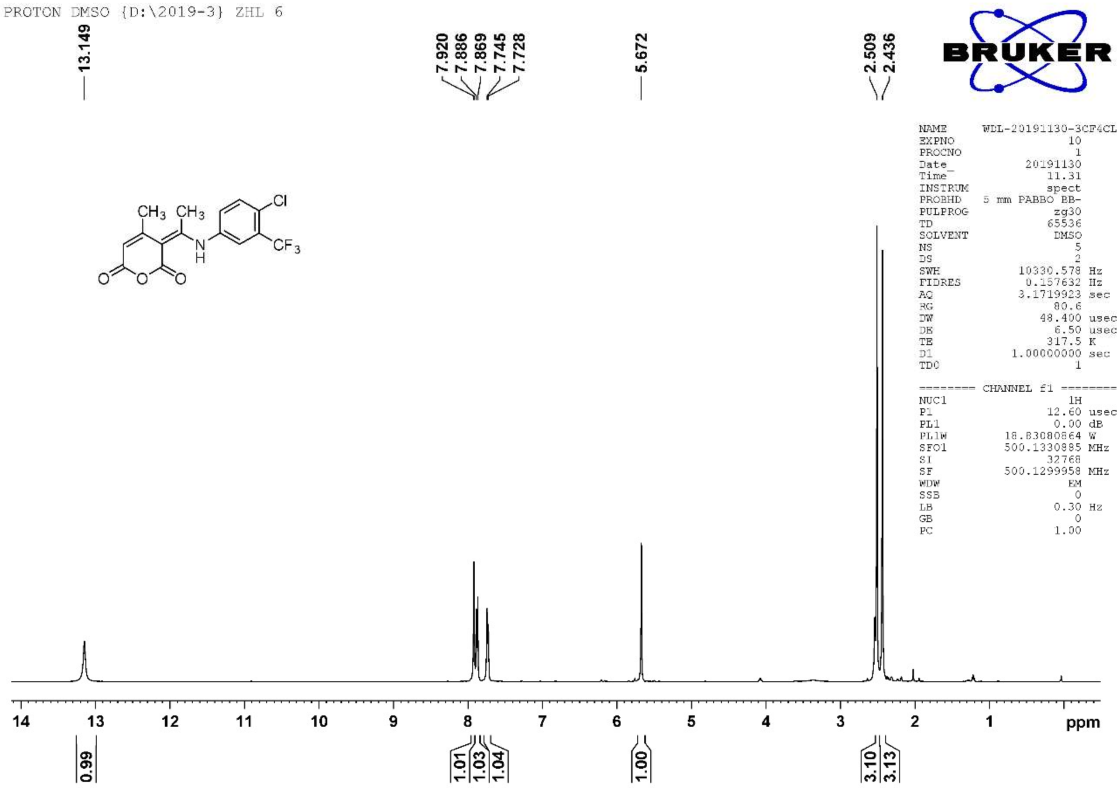

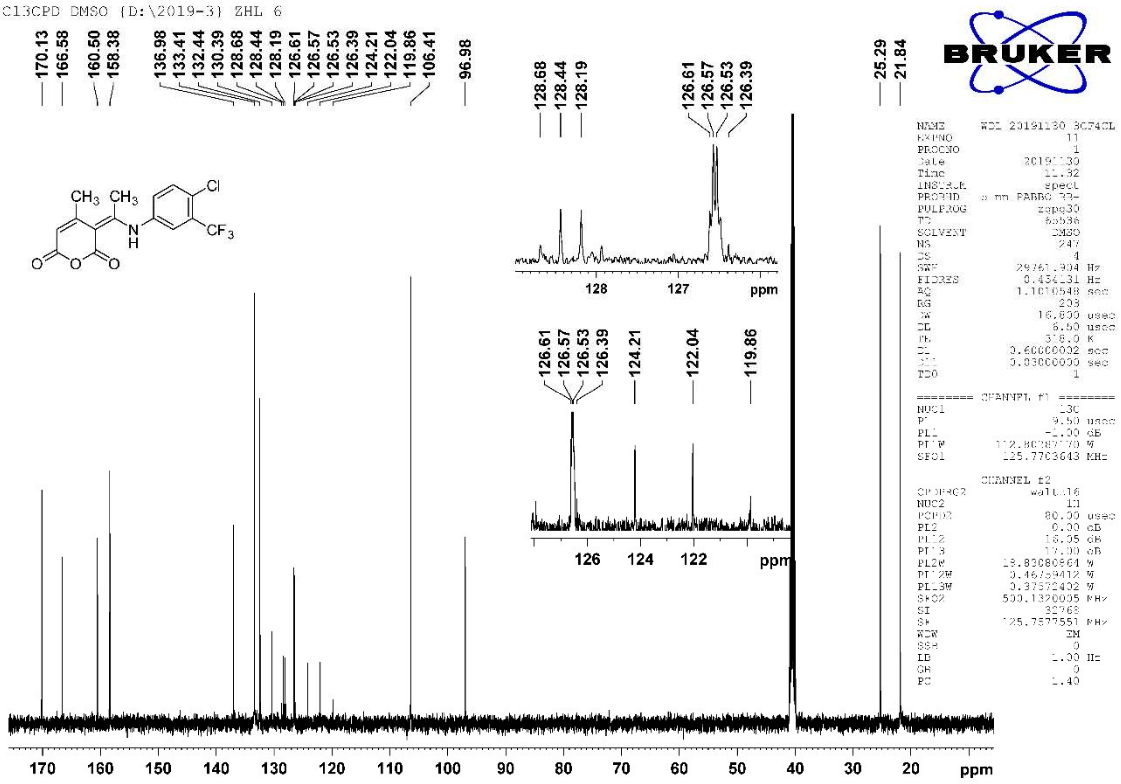

^{1}H and ^{13}C-NMR spectra of compound 4p.

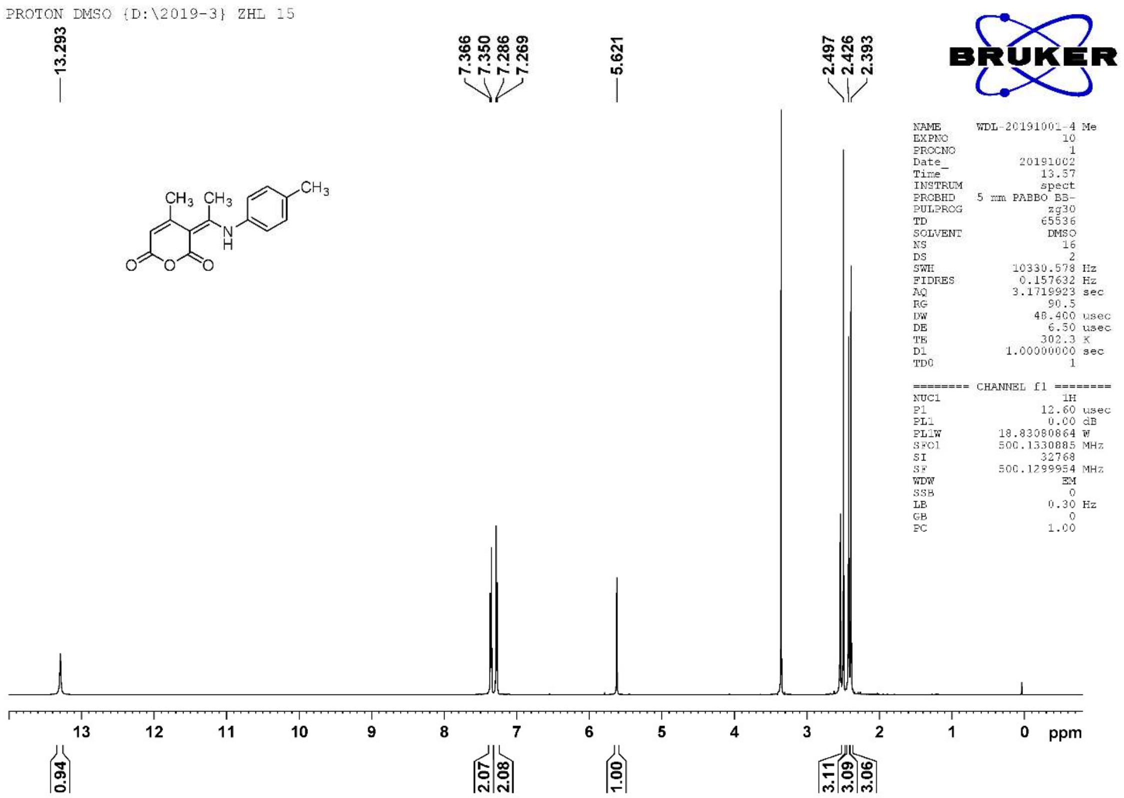

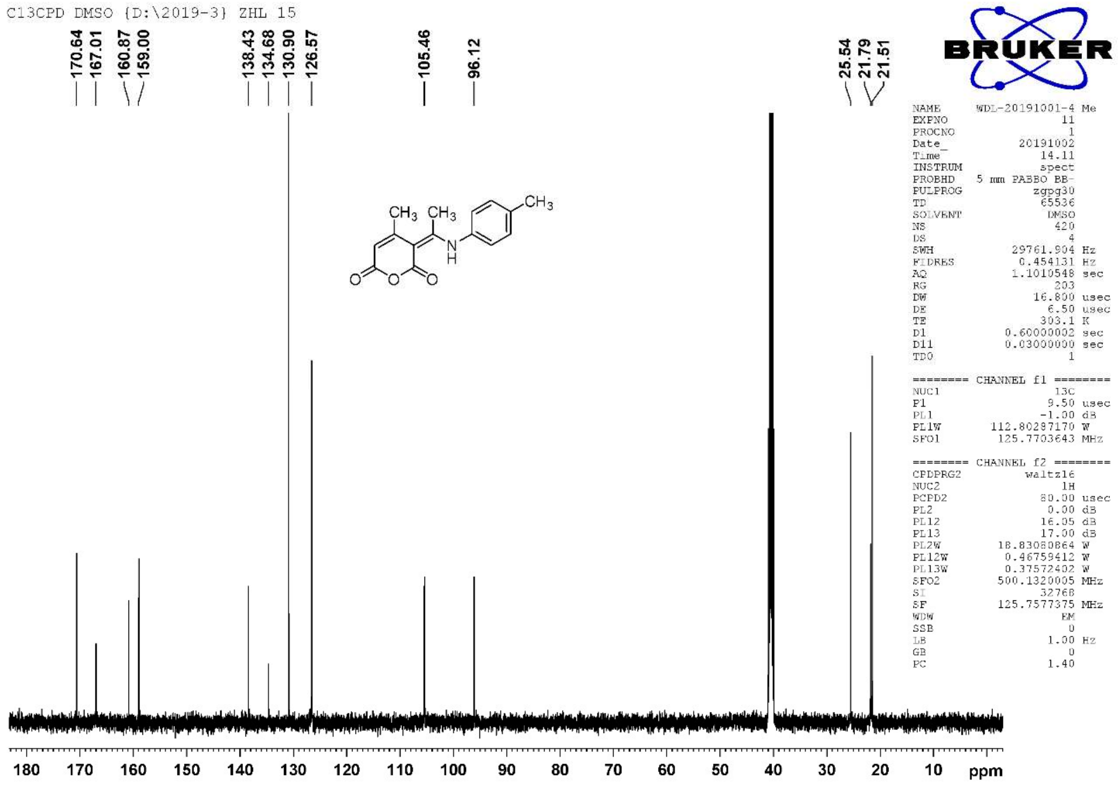

^{1}H and ^{13}C-NMR spectra of compound 4q.

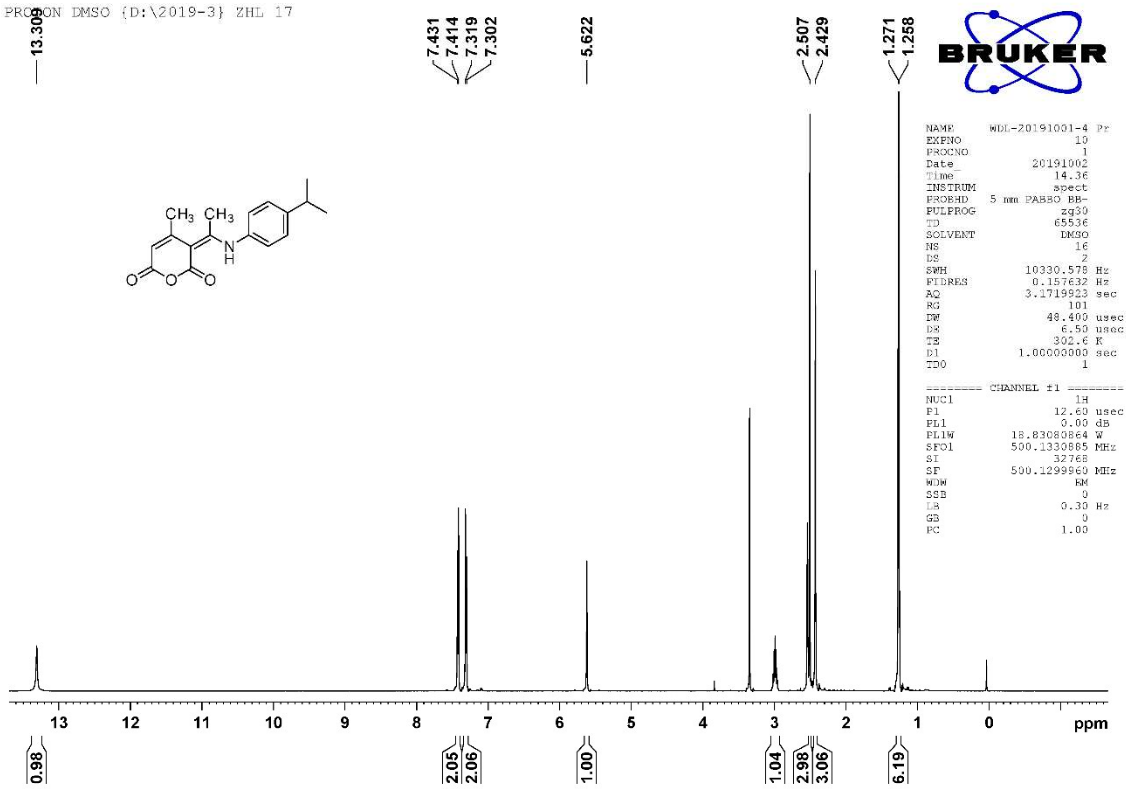

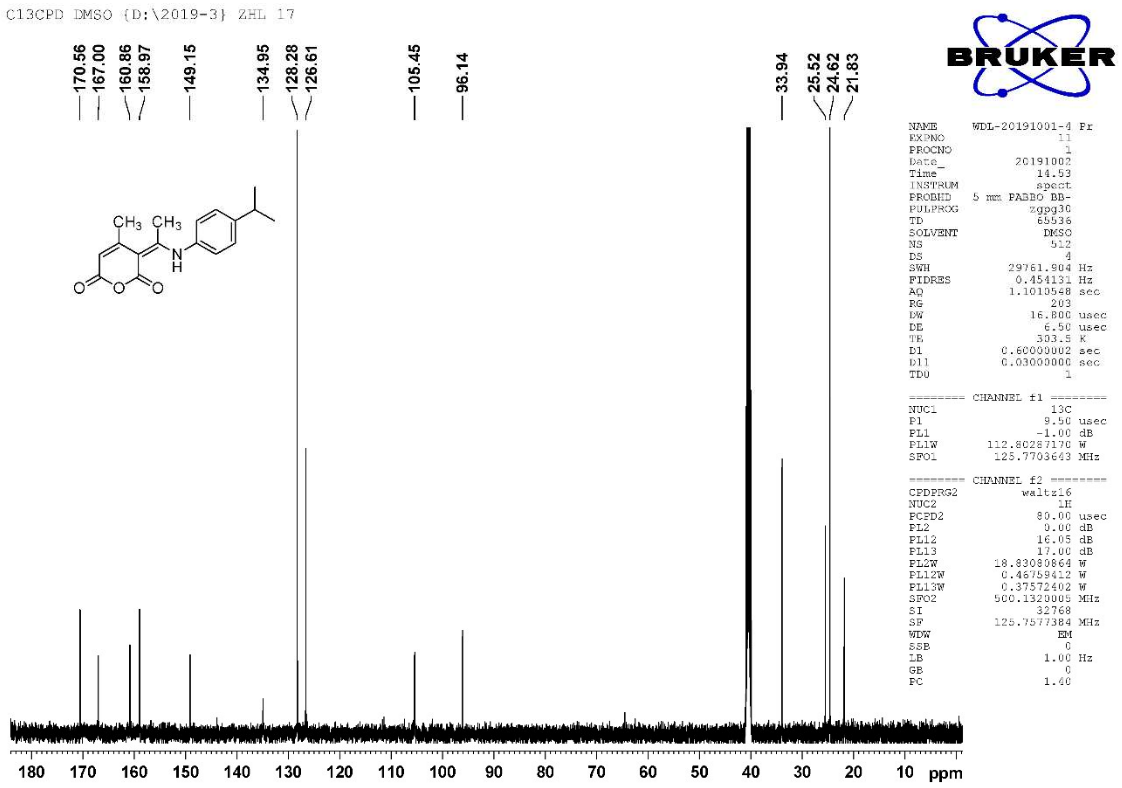

^{1}H and ^{13}C-NMR spectra of compound 4r.

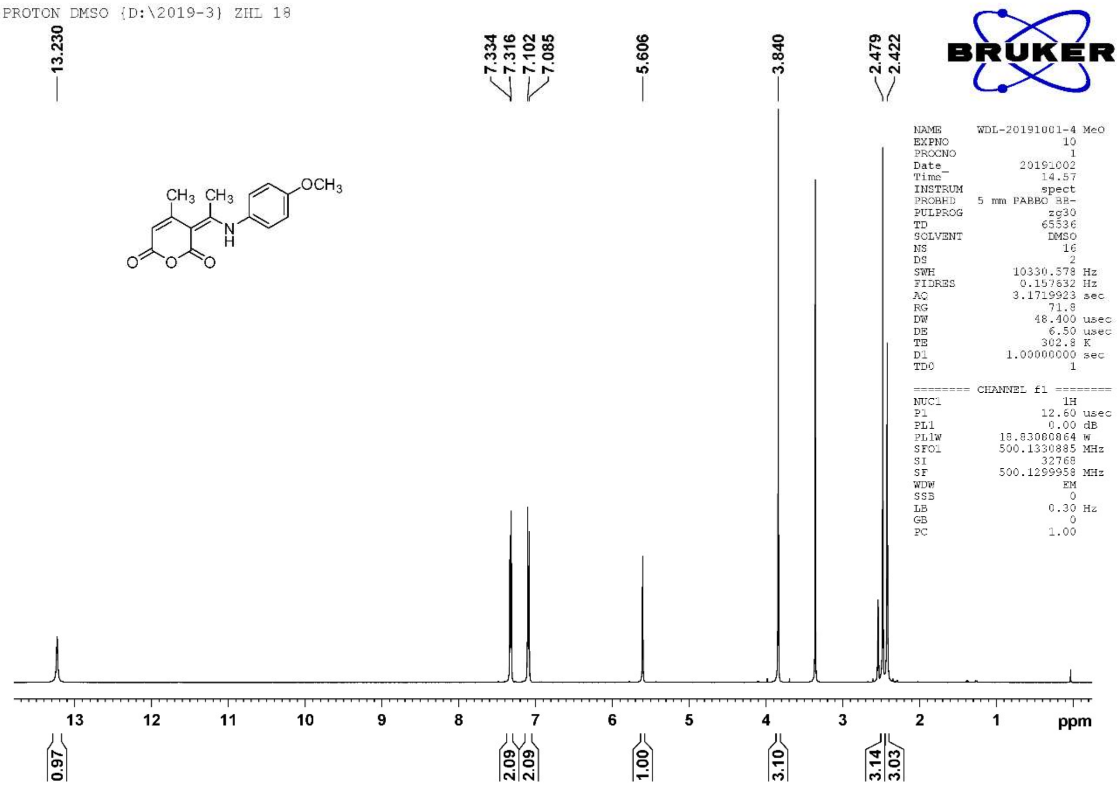

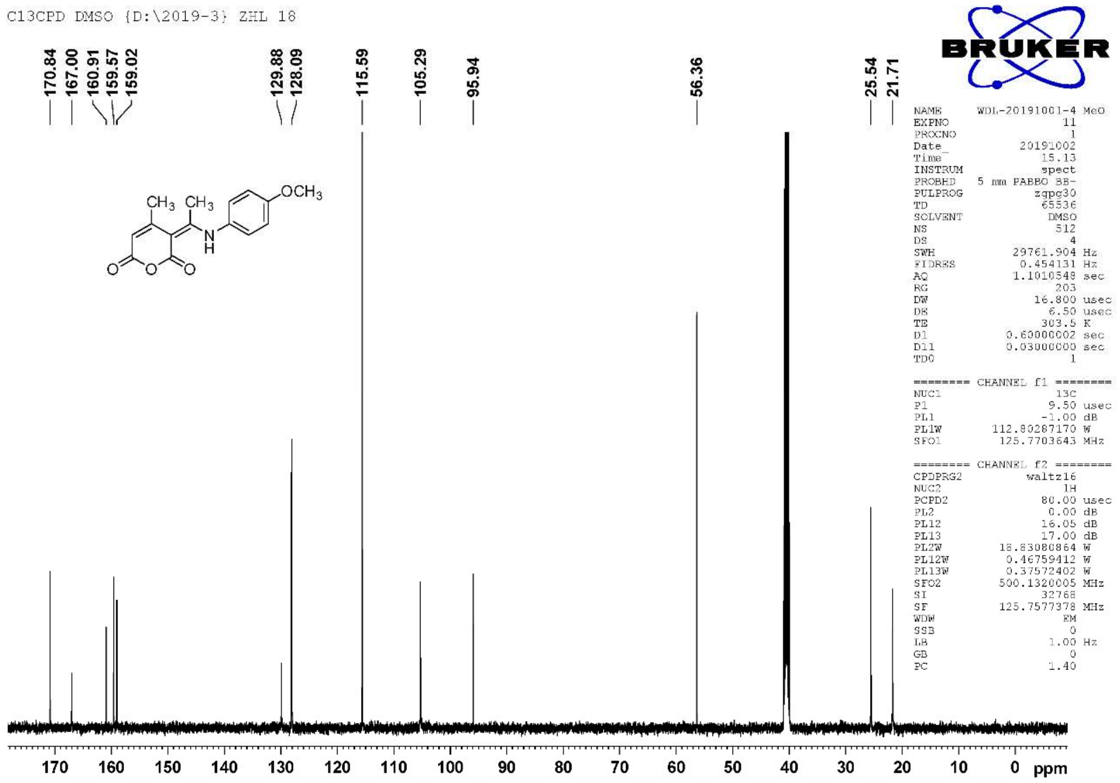

^{1}H and ^{13}C-NMR spectra of compound 4s.

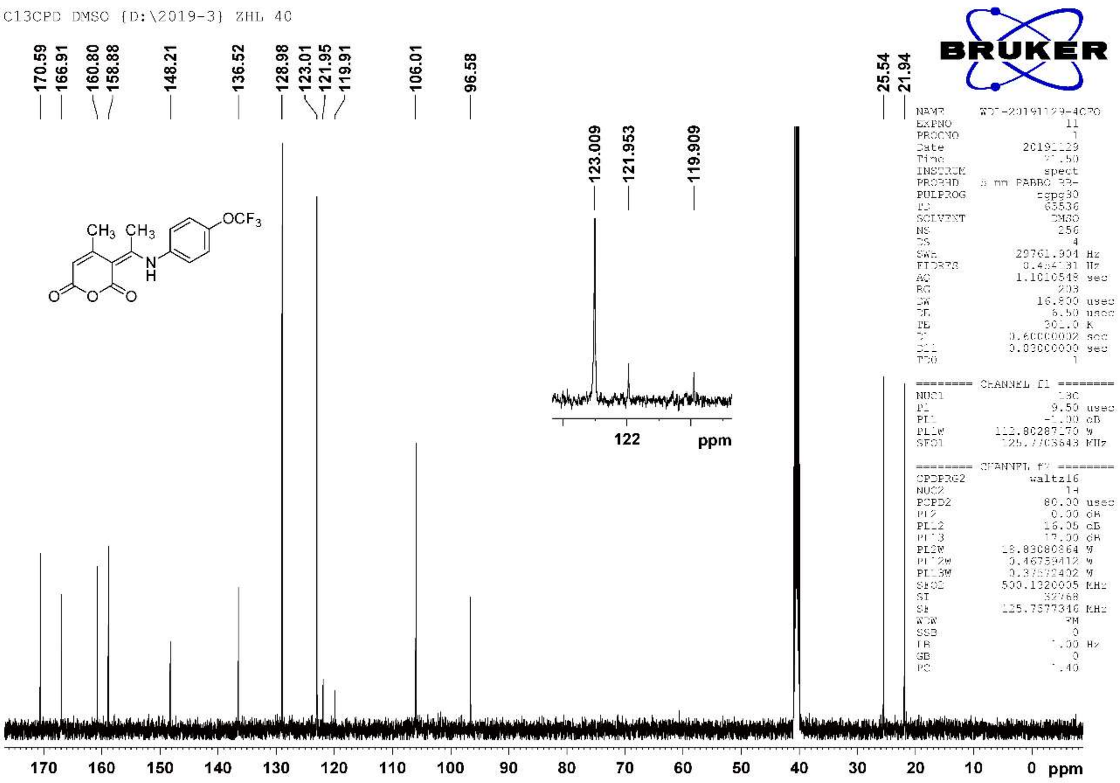

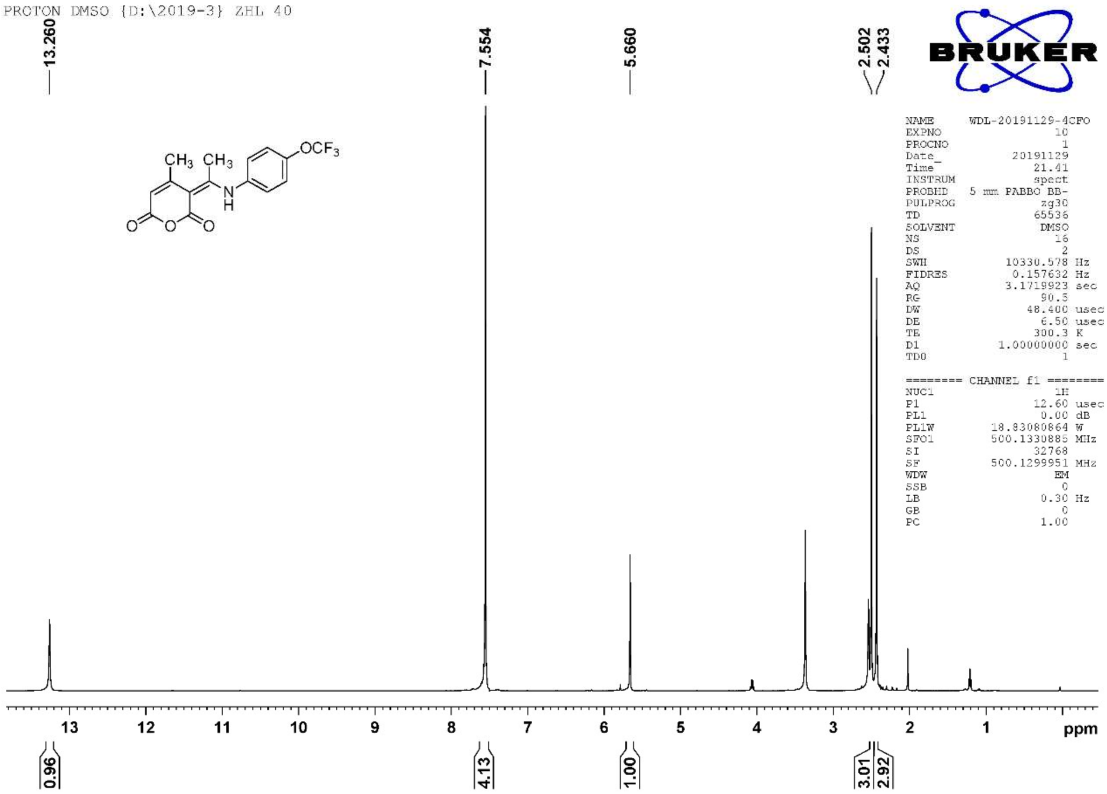

^{1}H and ^{13}C-NMR spectra of compound 4t.

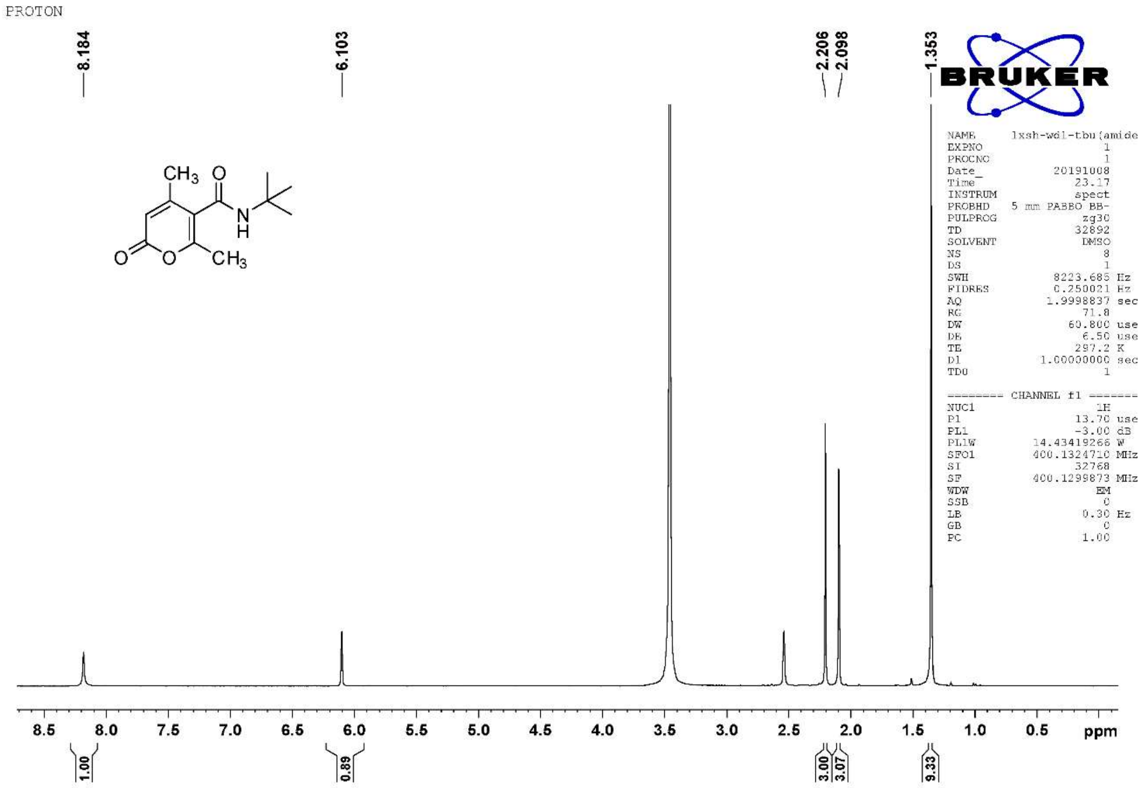

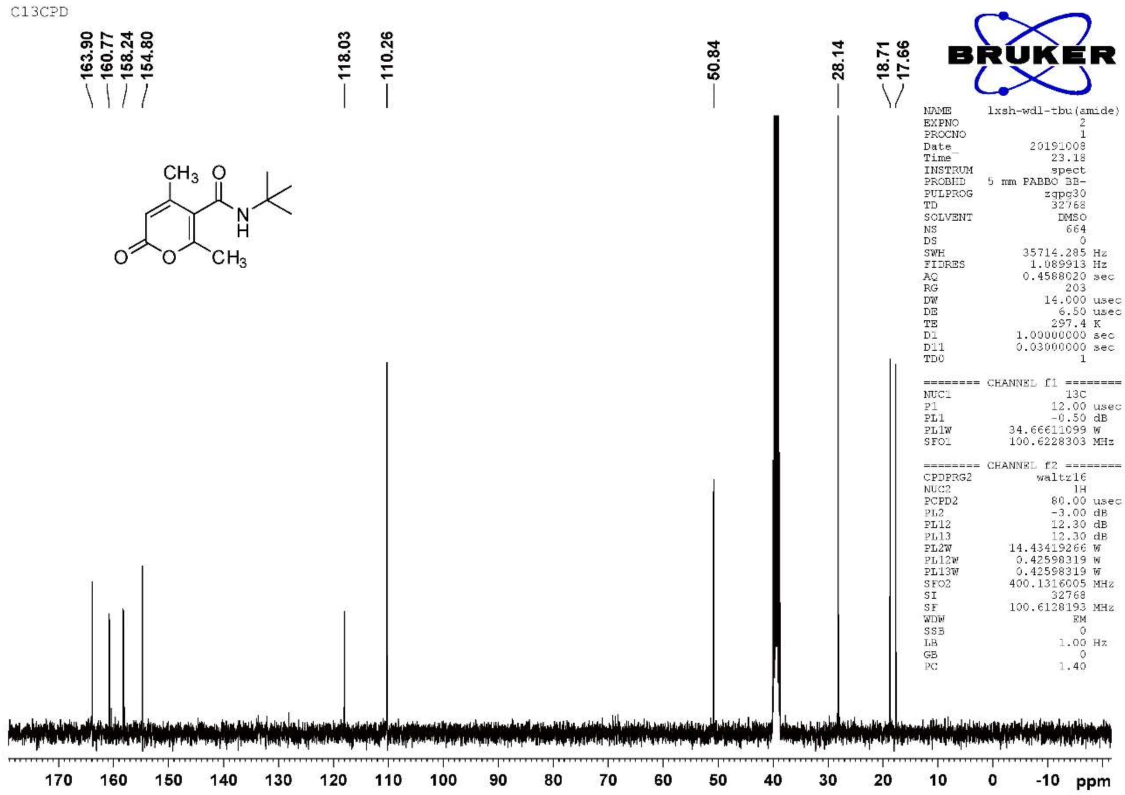

^1H and ^{13}C-NMR spectra of compound 4u.

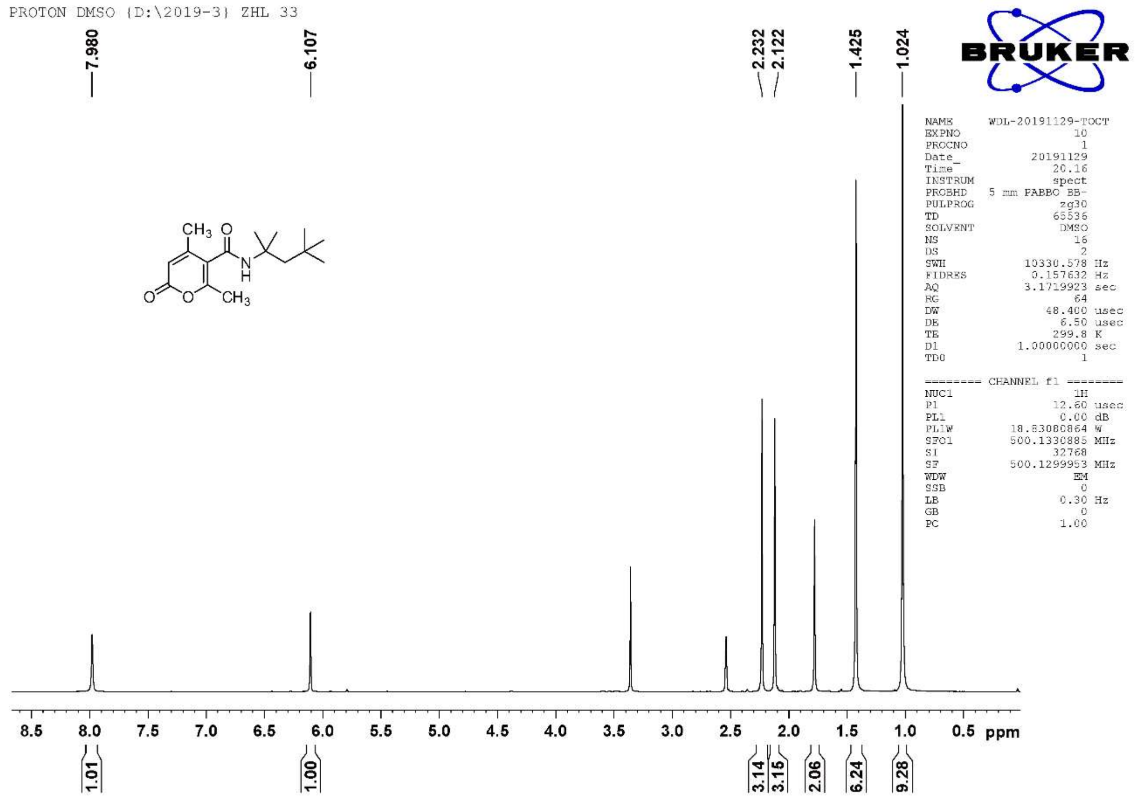

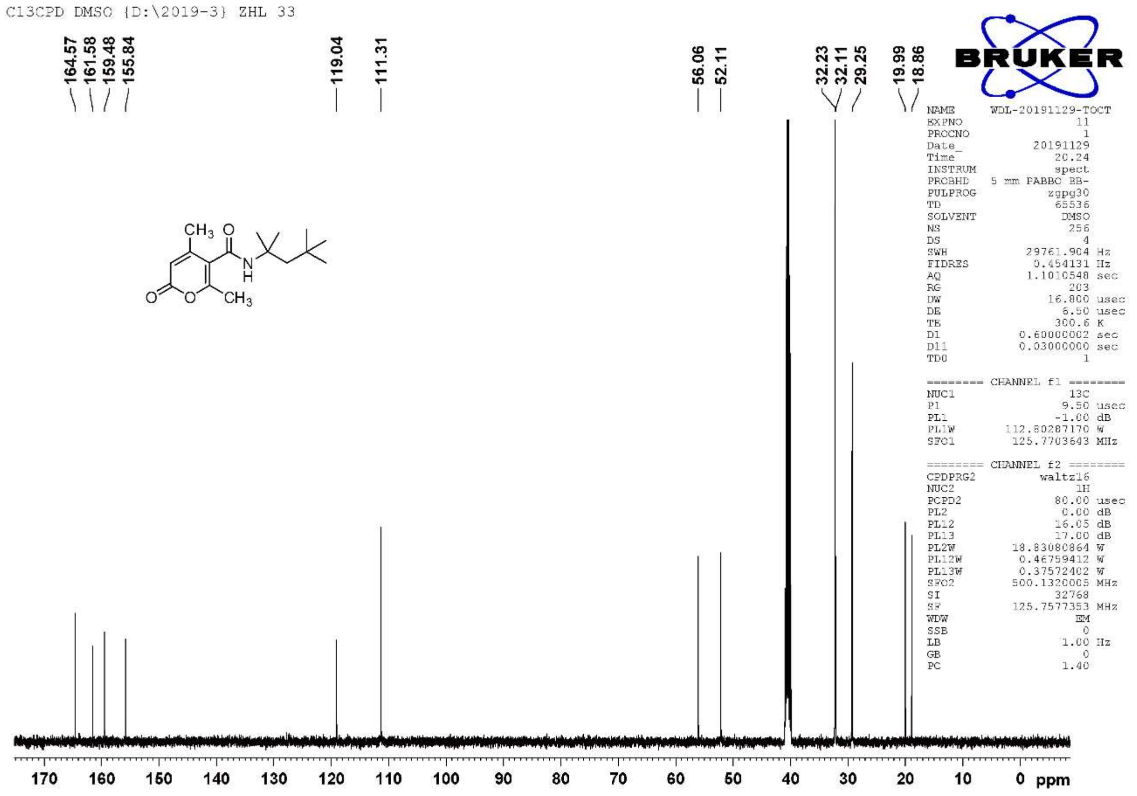

^{1}H and ^{13}C-NMR spectra of compound 4v.

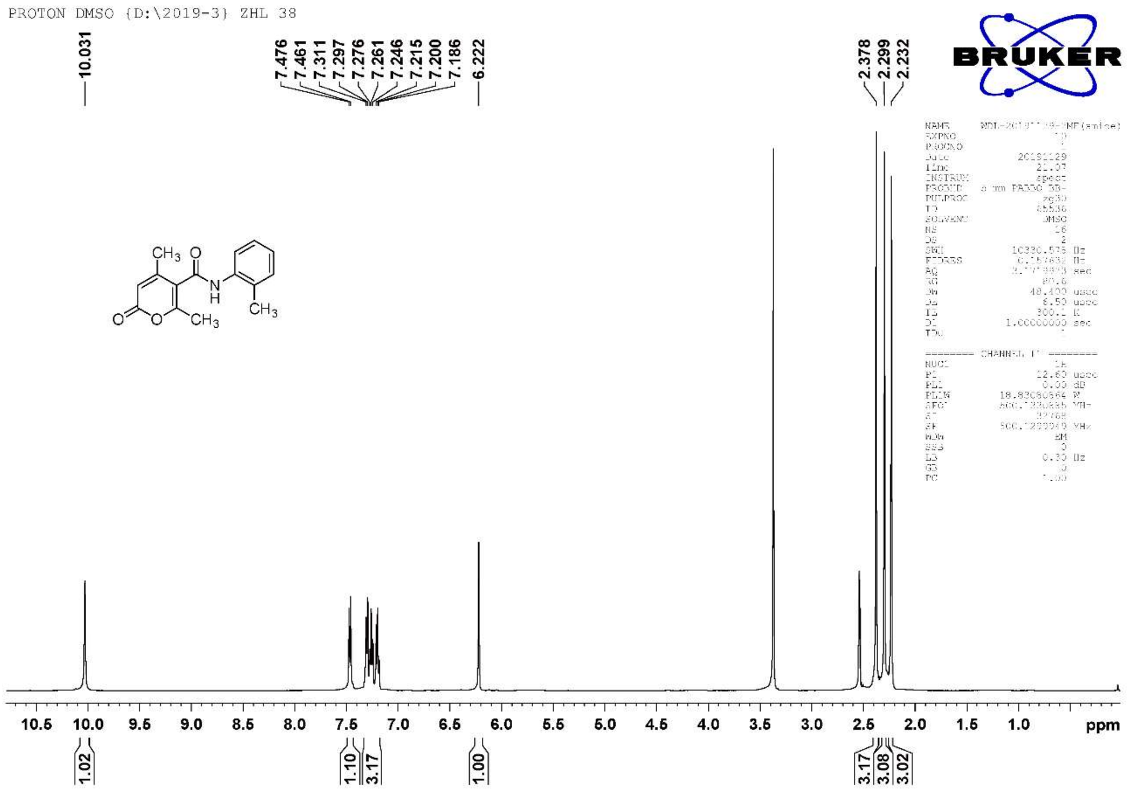

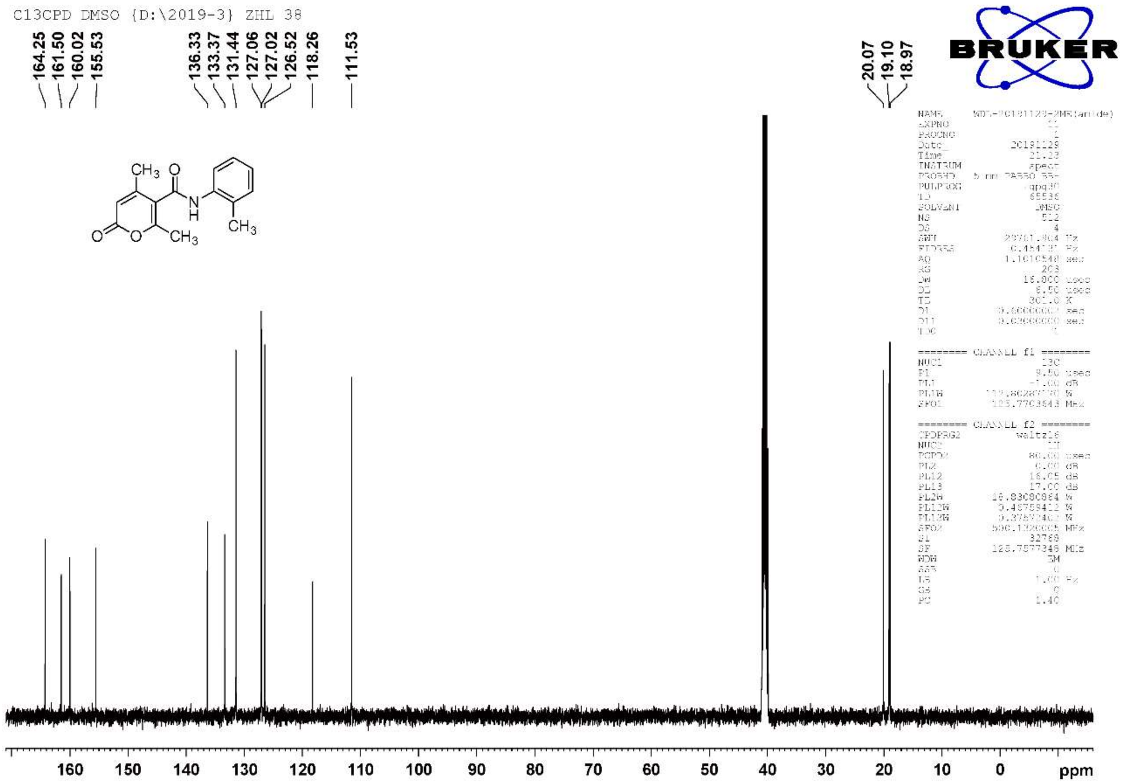

^{1}H and ^{13}C-NMR spectra of compound 4w.

2. Computational details

The structure of ketene intermediate was optimized using DFT method at B3LYP/6-31+G** level by GAUSSIAN09 packages software [1]. The atom charges of ketene intermediate at this level were calculated [2,3].

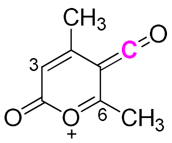

Figure S1. The structure of ketene intermediate.

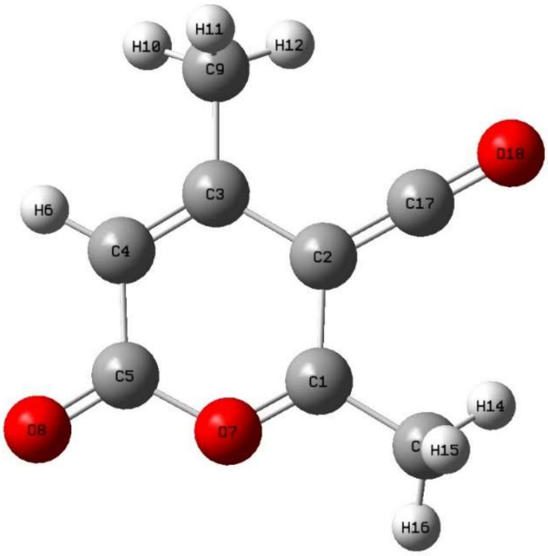

Figure S2. The atom numbers of ketene intermediate in calculation.

Table S1. Summary of Natural Population Analysis (NPA) charges of ketene intermediate.

Atom	No	Natural Charge
C	1	0.49573
C	2	-0.25324
C	3	0.12166
C	4	-0.11229
C	5	0.38621
H	6	0.09411
O	7	-0.17883
O	8	-0.15332
C	9	-0.16582
H	10	0.08726
H	11	0.08383
H	12	0.06306
C	13	-0.20595
H	14	0.09637
H	15	0.11923
H	16	0.11670
C	17	0.48691
O	18	-0.08161

Table S2. Mulliken charges of ketene intermediate.

Atom	No	Charge
C	1	0.351069
C	2	-0.09113
C	3	0.067323
C	4	-0.08082
C	5	0.309976
H	6	0.130896
O	7	-0.16222
O	8	-0.1223
C	9	-0.19989
H	10	0.110341
H	11	0.106052
H	12	0.084427
C	13	-0.2101
H	14	0.12032
H	15	0.14246
H	16	0.142814
C	17	0.338582
O	18	-0.0378

1 Gaussian 09, Revision A.02, M. J. Frisch, G. W. Trucks, H. B. Schlegel, G. E. Scuseria, M. A. Robb, J. R. Cheeseman, G. Scalmani, V. Barone, G. A. Petersson, H. Nakatsuji, X. Li, M. Caricato, A. Marenich, J. Bloino, B. G. Janesko, R. Gomperts, B. Mennucci, H. P. Hratchian, J. V. Ortiz, A. F. Izmaylov, J. L. Sonnenberg, D. Williams-Young, F. Ding, F. Lipparini, F. Egidi, J. Goings, B. Peng, A. Petrone, T. Henderson, D. Ranasinghe, V. G. Zakrzewski, J. Gao, N. Rega, G. Zheng, W. Liang, M. Hada, M. Ehara, K. Toyota, R. Fukuda, J. Hasegawa, M. Ishida, T. Nakajima, Y. Honda, O. Kitao, H. Nakai, T. Vreven, K. Throssell, J. A. Montgomery, Jr., J. E. Peralta, F. Ogliaro, M. Bearpark, J. J. Heyd, E. Brothers, K. N. Kudin, V. N. Staroverov, T. Keith, R. Kobayashi, J. Normand, K. Raghavachari, A. Rendell, J. C. Burant, S. S. Iyengar, J. Tomasi, M. Cossi, J. M. Millam, M. Klene, C. Adamo, R. Cammi, J. W. Ochterski, R. L. Martin, K. Morokuma, O. Farkas, J. B. Foresman, and D. J. Fox, Gaussian, Inc., Wallingford CT, 2016.

2 Li, L.; Wu, C.; Wang, Z.; Zhao, L.; Li, Z.; Sun, C.; Sun, T. Density functional theory (DFT) and natural bond orbital (NBO) study of vibrational spectra and intramolecular hydrogen bond interaction of L-ornithine-L-aspartate. *Spectrochim. Acta A* **2015**, *136*, 338–346.

3 Katritzky, A.R.; Duell, B.L.; Gallos, J.K.; Durst, H.D. ^{13}C NMR study of 2-iodoso- and 2-iodoxy-benzoic acids and their sodium salts. *Mag. Reson. Chem.* **2007**, *45*, 532–543.
